# Physalin F Promotes AFG3L2-Mediated Degradation of VISA/MAVS to Suppress Innate Immune Response to RNA Virus

**DOI:** 10.3390/pathogens15010074

**Published:** 2026-01-09

**Authors:** Xiao-Nan Gao, Hong-Bing Shu, Mi Li

**Affiliations:** Department of Infectious Diseases, Medical Research Institute, Zhongnan Hospital of Wuhan University, Wuhan University, Frontier Science Center for Immunology and Metabolism, State Key Laboratory of Virology and Biosafety, Taikang Center for Life and Medical Sciences, Wuhan University, Wuhan 430071, China; xn_gao@whu.edu.cn

**Keywords:** physalin F, VISA/MAVS, AFG3L2, innate immunity, virus

## Abstract

Upon RNA virus infection, viral RNA is sensed by the RIG-I-like receptors (RLRs), which signal through the adaptor protein VISA/MAVS to induce an innate antiviral response. How the VISA-mediated innate antiviral response is regulated and whether it can be targeted for drug development against diseases caused by RNA virus infection needs to be further investigated. Here we report that physalin F, a natural secosteroid isolated from *Physalis angulata* L., inhibits innate immune response to RNA virus. Mechanistically, physalin F binds to and promotes the activation of the mitochondrial *m*-AAA protease AFG3L2, which subsequently mediates the degradation of VISA. Knockdown of AFG3L2 promotes RLR-mediated innate antiviral signaling, whereas physalin F inhibits innate immune response to RNA virus both in cells and mice. Our study discovers physalin F as an inhibitor of VISA-mediated innate antiviral response as well as a candidate compound for the treatment of related diseases. More importantly, our findings suggest that AFG3L2 constitutively mediates degradation of VISA under physiological conditions, which represents a novel negative regulatory mechanism of RLR-mediated innate antiviral response.

## 1. Introduction

The innate immune response serves as the first line of host defense against microbial pathogens, which is initiated by recognition of conserved microbial components, such as their nucleic acids, by the host pattern recognition receptors (PRRs). The sensing of microbial components by PRRs triggers signaling pathways that eventually lead to the induction of type I interferons (IFNs), pro-inflammatory cytokines, and other anti-microbial effector genes, leading to innate antiviral response and promotion of late adaptive immune response [1,2,3,4].

Upon infection of an RNA virus, the viral RNA is detected by the RIG-I-like receptors (RLRs), which include retinoic acid inducible gene-I (RIG-I) and melanoma differentiation-associated gene 5 (MDA5) [5,6]. Upon binding to viral RNA, RLRs undergo a conformational rearrangement and expose their N-terminal CARD domains, which interact with the CARD domain of the Virus-Induced Signaling Adaptor (VISA, also known as MAVS and IPS-1) that is anchored on the outer membrane of mitochondria [7,8,9]. This leads to the redistribution of VISA on the mitochondrial membrane and formation of large prion-like VISA aggregates [10], which in turn recruit TRAF family ubiquitin E3 ligases to catalyze the formation of polyubiquitin chains to activate the downstream kinases IKK and TBK1, leading to activation of the transcription factors NF-κB and IRF3, and induction of downstream antiviral effector genes [11,12,13].

As the central adaptor in the RLR-signaling pathway, the stability and activity of VISA are tightly regulated. Several proteins, including sorting nexin 8 (SNX8) [14] and TRIM31 [15] have been shown to promote VISA aggregation and activation, whereas G patch domain-containing protein 3 (GPATCH3) [16] and Thioredoxin 2 (TRX2) [17] impair the assembly of VISA-associated complexes and therefore negatively regulate RLR-mediated signaling. Furthermore, multiple E3 ubiquitin ligases, including RNF5 [18], MARCH5 [19,20], and Smurf2 [21] catalyze K48-linked polyubiquitination of VISA, targeting it for proteasomal degradation and thus suppressing RLR-mediated innate antiviral signaling.

De-regulation of innate immune signaling causes various autoinflammatory or immunodeficient diseases, such as systemic lupus erythematosus (SLE) and Aicardi Goutieres syndrome (AGS) [22,23]. Current therapies for immune disorders are usually associated with multiple adverse effects, such as diabetes mellitus, cataracts, osteoporosis, increased susceptibility to infections, and mood disorders [24,25]. Natural products derived from plants, fungi, and microorganisms represent a class of promising drugs for human diseases [26], and certain small molecules exhibit potent immunomodulatory effects [27]. For example, aspirin has been shown to reduce DNA-mediated autoimmune response in both murine models and cells from AGS patients [28]. Epigallocatechin gallate (EGCG) extracted from green tea inhibits DNA-induced IFN production by disrupting the G3BP1-cGAS complexes [29].

To identify potential small molecule inhibitors of virus-induced expression of antiviral viral effecter genes, such as type I IFNs, we screened a library of natural small-molecule compounds and identified physalin F as an effective inhibitor for RNA virus-triggered induction of type I IFN genes. Physalin F is a secosteroid isolated from *Physalis angulata* L., a Solanaceae plant historically used in traditional medicine to treat inflammatory conditions [30]. It has been shown that physalin F has significant immunomodulatory activity by reducing the production of key cytokines such as IL-2, IL-4, IL-10, and IFN-γ in activated splenocytes and inhibiting lymphocyte proliferation [31,32]. Physalin F also induces apoptotic cell death of lymphocytes derived from human-T lymphotropic virus type 1 (HTLV-1) patients [33]. In addition, physalin F appears to be a promising anti-cancer agent by triggering apoptosis of breast cancer cells [34]. Additionally, physalin F induces significant cytotoxicity in human renal carcinoma cells (A498, ACHN, and UO-31) through a reactive oxygen species (ROS)-mediated mitochondrial pathway that inhibits the activation of NF-κB [35].

In this study, we found that physalin F directly binds to AFG3-like matrix AAA peptidase subunit 2 (AFG3L2), a subunit of the mitochondrial ATPase-associated with diverse cellular activities (*m*-AAA) protease [36]. The binding of physalin F to AFG3L2 increases its protease activity, which promotes the degradation of VISA, leading to inhibition of RLR-mediated innate immune response. Our findings reveal AFG3L2-mediated degradation of VISA as a new regulatory mechanism of RLR-mediated innate immune response. Additionally, the identification of physalin F and its target AFG3L2 provides a new strategy for the development of therapeutics for RLR-driven inflammatory diseases.

## 2. Materials and Methods

### 2.1. Reagents, Antibodies, Viruses, and Cells

Physalin F (TargetMol, Wellesley Hills, MA, USA, T8716); Dual-Specific Luciferase Assay Kit (Promega, Madison, WI, USA, E2490); puromycin (Thermo Fisher Scientific, Waltham, MA, USA); M-MLV reverse transcriptase (Invitrogen, Carlsbad, CA, USA, 28025-013); SYBR (Bio-Rad laboratories, Hercules, CA, USA); RiboLock RNase inhibitor, pyrophosphatase (Thermo Fisher Scientific, Waltham, MA, USA); protease inhibitor cocktail (Roche, Basel, Switzerland); polybrene (Millipore, Burlington, MA, USA); Clarity™ Western ECL Substrate (Bio-Rad, Hercules, CA, USA); ELISA kits for murine IFN-β (PBL Assay Science, Piscataway, NJ, USA, 42400), murine IL-6 (BioLegend, San Diego, CA, USA, 431304); mouse antibodies against HA (OriGene, Rockville, MD, USA, TA180128) and FLAG (Sigma-Aldrich, Burlington, MA, USA, F3165); mouse antibodies against β-Tubulin (ABclonal, Woburn, MA, USA, A12289); rabbit antibodies against FLAG (14793), Rig-I (3743), β-actin (5125), p-IRF3^S396^ (4947), STAT1 (14994), p-STAT1^Y701^ (9167) STAT2 (72604), and p-STAT2^Y690^ (88410) (Cell Signaling Technology, Danvers, MA, USA); rabbit antibodies against TBK1 (ab40676), p-TBK1^S172^ (ab109272), and p-IRF3^S386^ (ab76493) (Abcam, Cambridge, UK); rabbit antibodies against AFG3L2 (14631-1-AP), TOM40 (18409-1-AP), TOM70 (14528-1-AP) (Proteintech, Rosemont, IL, USA), VISA (Bethyl Laboratories, Montgomery, TX, USA, A300-782A), and GFP (GeneTex, Irvine, CA, USA, GTX113617); and HRP-conjugated anti-FLAG monoclonal antibody (Sigma-Aldrich, A8592) were purchased from the indicated companies. Rabbit antisera against murine IRF3 were raised using the full length recombinant murine IRF3 protein as an antigen.

Sendai virus (SeV) and vesicular stomatitis virus (VSV) were previously described by our laboratory [37].

HEK293T cells were originally provided by Dr. Gary Johnson (National Jewish Health, Denver, CO, USA) and cultured in Dulbecco’s Modified Eagle’s Medium (Gibco, Grand Island, NY, USA) supplemented with 10% fetal bovine serum (FBS, CellMax), 1% penicillin-streptomycin (HyClone, Logan, UT, USA) at 37 °C with 5% CO_2_. THP 1 cells were obtained from the American Type Culture Collection (Manassas, VA, USA) and cultured in RPMI 1640 (Gibco) supplemented with 10% FBS, 1% penicillin-streptomycin at 37 °C with 5% CO_2_.

### 2.2. Constructs

The ISRE luciferase plasmid, as well as mammalian expression plasmids for FLAG-tagged RIG I, VISA, TBK1, IRF3, STAT1, and STAT2, were previously described [38,39]. Mammalian expression plasmids for FLAG-tagged HSPD1, EEF1A1, ACTC1, SODM, GSR, RPN2, ATP5F1B, PREB, CLTC, DDOST, and PSAT1; HA-tagged AFG3L2 and its mutants; HA-tagged ACTG1, SLC25A5, TFAM, and HSP90AA1 were constructed via standard molecular biology techniques. Guide RNA (gRNA) plasmids targeting AFG3L2 were constructed in a lenti-CRISPR-V2 vector, which was provided by Dr. Shu-Wen Wu (Wuhan University, Wuhan, China).

### 2.3. Transfection and Reporter Assay

HEK293T cells were transfected using calcium phosphate precipitation. The empty control plasmid was added to ensure that each transfection receives the same amount of total DNA. In reporter assays, a pRL-TK (Renilla luciferase) reporter plasmid (0.01 μg) was added to each transfection for normalization of transfection efficiency. Luciferase assays were performed using a Dual-Specific Luciferase Assay Kit (Promega, Madison, WI, USA). Firefly luciferase activities were normalized based on Renilla luciferase activities. The IC_50_ value was determined from the sigmoid dose–response curve using the GraphPad Prism 9 (GraphPad Software, San Diego, CA, USA).

### 2.4. Screening of Compounds

The library, consisting of 903 compounds were obtained from TargetMol. The final concentration of compounds used in the experiments was 5 μM. HEK293T cells stably expressing ISRE-luciferase reporter were seeded into 96-well plates at a density of 5 × 10^4^ per well and incubated overnight. The cells were treated with compounds (5 μM) for 30 min and infected with SeV for 8 h before luciferase reporter assays.

### 2.5. RT-qPCR

Total RNA was isolated from cells for RT-qPCR analysis to measure the mRNA abundance of specific genes. The data shown are the relative abundance of the indicated mRNA normalized to the level of *GAPDH* mRNA. Gene-specific primer sequences were shown as follows:

Human *GAPDH*: 5′-GTCTCCTCTGACTTCAACAGCG-3′ (forward) and 5′-ACCACCCTGTTGCTGTAGCCAA-3′ (reverse);

Human *IFNB1*: 5′-CTTGGATTCCTACAAAGAAGCAGC-3′ (forward) and 5′-TCCTCCTTCTGGAACTGCTGCA-3′ (reverse);

Human *ISG56*: 5′-GCCTTGCTGAAGTGTGGAGGAA-3′ (forward) and 5′-ATCCAGGCGATAGGCAGAGATC-3′ (reverse);

Human *IFI44*: 5′-GTGAGGTCTGTTTTCCAAGGGC-3′ (forward) and 5′-CGGCAGGTATTTGCCATCTTTCC-3′ (reverse);

Human *CXCL10*: 5′-GGTGAGAAGAGATGTCTGAATCC-3′ (forward) and 5′-GTCCATCCTTGGAAGCACTGCA-3′ (reverse);

Murine *Gapdh*: 5′-ACGGCCGCATCTTCTTGTGCA-3′ (forward) and 5′-ACGGCCAAATCCGTTCACACC-3′ (reverse);

Murine *Ifnb1*: 5′-TCCTGCTGTGCTTCTCCACCACA-3′ (forward) and 5′-AAGTCCGCCCTGTAGGTGAGGTT-3′ (reverse);

Murine *Isg56*: 5′-ACAGCAACCATGGGAGAGAATGCTG-3′ (forward) and 5′-ACGTAGGCCAGGAGGTTGTGCAT-3′ (reverse);

Murine *Cxcl10*: 5′-ATCATCCCTGCGAGCCTATCCT-3′ (forward) and 5′-GACCTTTTTTGGCTAAACGCTTTC-3′ (reverse).

### 2.6. CRISPR/Cas9 Gene Editing

We have used the CRISPR-Cas9 method for gene editing [40,41]. Briefly, double-stranded oligonucleotide sequences designed to target specific genes were inserted into the lenti-CRISPR-V2 vector. This plasmid was subsequently co-transfected with the packaging plasmids psPAX2 and the envelope plasmid pMD2.G into HEK293 cells. Two days after transfection, the recombinant viruses were collected to infect HEK293T cells in the presence of polybrene (8 μg/mL). Selection of gene-edited cells was performed using puromycin (1 μg/mL) over a period of at least 6 days to establish stable lines. The following oligonucleotides were used to construct the respective gRNA plasmids:

g*NC*: 5′-GTAGTCGGTACGTGACTCGT-3′;

g*AFG3L2*#1: 5′-GAATGAGACTCACTCTAGCA-3′;

g*AFG3L2*#2: 5′-AAGGCGATGATGAGCACCGT-3′.

### 2.7. Immunoblotting Analysis

Cells were harvested and washed twice with ice-cold PBS, followed by lysed with 2 × SDS loading buffer (100 mM Tris-HCl [pH 6.8], 4% SDS, 0.02% bromophenol blue, 20% glycerol, and 2% β-mercaptoethanol). The lysates were boiled at 95 °C for 15 min. Samples were separated by 10% SDS-PAGE and transferred onto nitrocellulose (NC) membranes (Millipore, Burlington, MA, USA). The membranes were blocked with 5% non-fat milk in TBST (Tris-buffered saline containing 0.1% Tween-20) for 1 h at room temperature, then incubated with primary antibodies overnight at 4 °C. The membranes were then washed three times with TBST and incubated with HRP-conjugated secondary antibodies for 1 h at room temperature. Protein bands were visualized using an Enhanced Chemiluminescence (ECL) detection system and exposed to X-ray films in a darkroom.

### 2.8. Mice

C57BL/6 mice were maintained in specific-pathogen-free rooms in the Medical Research Institute at Wuhan University. Six-week-old female mice were randomly allocated to each experimental group, and littermates were used as controls. All animal experiments were performed in accordance with the Wuhan University Medical Research Institute Animal Care and Use Committee guidelines.

### 2.9. ELISA

Mice were treated by oral route with DMSO or physalin F (30 mg/kg) for half an hour, and then intraperitoneally injected with VSV or SeV (2 × 10^8^ pfu per mouse) for 8 h before measurement of serum cytokine levels by ELISA assay.

### 2.10. Preparation of BMDMs and BMDCs

We prepared bone marrow-derived macrophages (BMDMs) and dendritic cells (BMDCs) as previously described [42]. Briefly, mouse bone marrow-derived monocytes were cultured for 3–5 days in the presence of 10% M-CSF conditional medium collected from L929 cell culture for differentiation into macrophages (BMDMs), or were cultured in medium containing murine GM-CSF (50 ng/mL) for 7 days for differentiation into dendritic cells (BMDCs).

### 2.11. Limited Proteolysis Coupled with Mass Spectrometry (LiP-MS)

LiP-MS assay was performed by SpecAlly Life Technology Co., Ltd. (Wuhan, China) as described [43]. Briefly, cells were lysed in lysis buffer (PBS with cocktail protease inhibitors, pH 7.4) and centrifuged at 12,000× *g* for 5 min. The cell lysates were incubated with DMSO or physalin F (10 μM) for 10 min at 25 °C, and then 1% proteinase K was added for 5 min at 25 °C. The reaction was stopped by heating at 98 °C for 3 min. Under physiological conditions, proteins are dynamic, and the cleavage sites of peptides would be exposed to proteases to generate structure-specific protein fragments. Fragments then undergo digestion with the sequence-specific enzyme trypsin, generating peptide mixtures appropriate for bottom-up proteomic analysis. Tris (2-carboxyethyl) phosphine (TCEP) and chloroacetamide (CAA) were added to each sample, followed by incubation at 37 °C for 1 h to perform the reductive alkylation reaction. A 100 mM Tris-HCl buffer was added to the sample to dilute the concentration of sodium deoxycholate (SDC) to below 1%. Trypsin was then added at a mass ratio of 1:50 (enzyme: protein), and the mixture was incubated with shaking at 37 °C overnight. Peptide analysis was performed using liquid chromatography coupled with tandem mass spectrometry (LC-MS/MS). All the data was subjected to a rigorous computational workflow, including data acquisition, peptide label-free quantitation, and screening for physalin F binding proteins.

### 2.12. Cellular Thermal Shift Assay (CETSA)

HEK293T cells were transfected with mammalian expression plasmids for the tested proteins for 16 h, and then exposed to DMSO or physalin F (10 μM) for 2 h before being suspended in PBS with 1 mM phenylmethylsulphonyl fluoride. The suspensions were divided into equal volumes and then subjected to thermal digestion at a range of temperatures for 3 min. After undergoing 3 freeze–thaw cycles in liquid nitrogen, separation of cell debris and aggregates from the soluble protein fraction was performed through centrifugation at 20,000× *g* for 20 min at 4 °C, and the supernatants were collected for immunoblotting analysis.

### 2.13. Statistical Analysis

Data analysis was performed using two-tailed unpaired Student’s t test or two-way ANOVA and Prism GraphPad software for statistical analyses. The number of asterisks represents the degree of significance with respect to the *p* value. Statistical significance was set to *p* < 0.05 * and *p* < 0.01 **.

## 3. Results

### 3.1. Identification of Physalin F as an Inhibitor of RNA Virus-Induced Innate Antiviral Response

**Figure 1:**
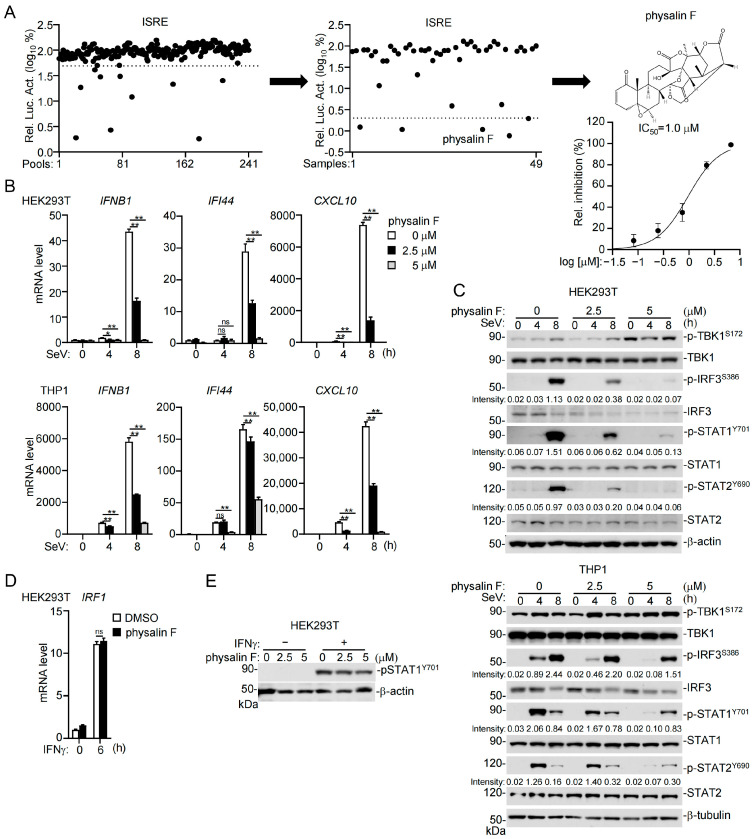
Identification of physalin F as an inhibitor of SeV-induced innate immune signaling. (**A**) Compounds of a natural small molecule library (*n* = 903) were sub-pooled into 4 compounds/sub-pool. HEK293T cells (5 × 10^4^) stably transduced with an ISRE luciferase reporter were treated with each of the sub-pools (5 μM for each compound) for 0.5 h, followed by infection with SeV for 8 h before luciferase assays (left dot plot, the dashed line indicates inhibition of SeV-induced ISRE activation by 50%). Forty-eight individual compounds from the 12 positive sub-pools (inhibition rate > 50%) were then tested for their effects on SeV-induced ISRE activation by reporter assays (middle dot plots, the dashed line indicates inhibition of SeV-induced ISRE activation by >98%). The arrows indicate the workflow. The candidate compound physalin F was further tested for its dose-dependent effects on SeV-induced ISRE activation by reporter assay. The IC_50_ value was calculated from the dose–response curve. IC_50_ = 1.0 μM, (95% CI: 0.8 to 1.1 μM). (**B**) Effects of physalin F on transcription of downstream genes induced by SeV in HEK293T and THP1 cells. HEK293T or THP 1 cells (1 × 10^6^) were treated with physalin F (0, 2.5, 5 μM) for 0.5 h and then infected with SeV for the indicated times before RT-qPCR analysis of mRNA levels of the indicated effector genes. *GAPDH* mRNA level was used as the internal control. (**C**) Effects of physalin F treatment on SeV-induced phosphorylation of TBK1, IRF3, STAT1, and STAT2. HEK293T or THP1 cells (1 × 10^6^) were treated with the indicated concentrations of physalin F for 0.5 h and then infected with SeV for the indicated times. Immunoblotting analysis was performed with the indicated antibodies. The relative band intensities, which are normalized to the corresponding β-actin bands, are quantitated by densitometry analysis using ImageJ (1.53c) software and shown under the blots. Original Western blot images can be found in Figure S1A. (**D**) Effects of physalin F on IFN-γ induced transcription of the IRF1 gene in HEK293T cells. HEK293T cells (1 × 10^6^) were treated with physalin F (0, 5 μM) for 0.5 h and then with IFN-γ for 6 h before RT-qPCR analysis of mRNA levels of the indicated antiviral genes. *GAPDH* mRNA level was used as the internal control. (**E**) Effects of physalin F on IFN-γ induced phosphorylation of STAT1. HEK293T (1 × 10^6^) were treated with physalin F (0, 2.5, 5 μM) for 0.5 h and then with IFN-γ for 6 h. Immunoblotting analysis was performed with the indicated antibodies. Original Western blot images can be found in Figure S1B. Data shown in (**A**) (dose experiment), (**B**,**D**) are mean ± SD; *n* = 3 technical replicates. ns, not significant, * *p* < 0.05; ** *p* < 0.01. Experiments in (**B**–**E**) were repeated at least two times with similar results.

To identify natural small molecule inhibitors of innate antiviral response, we screened a library consisting of 903 compounds, which were isolated and purified from plants, microorganisms, animals, and marine organisms. To do this, we examined the effects of these compounds (5 μM) on the activation of IFN-stimulated response element (ISRE) induced by infection of the RNA virus Sendai virus (SeV) in human embryonic kidney (HEK293T) cells by luciferase reporter assays [44,45]. In the primary screens, compounds were tested in pools of four, which identified 12 positive sub-pools that inhibited SeV-induced ISRE activation by 50% (Figure 1A, left dot plot, Table S1). Individual compounds from the 12 candidate pools were further screened by reporter assays, which identified five compounds, including physalin F, cucurbitacin B, tripterin, sanguinarine, and valepotriate, that could inhibit SeV-induced ISRE activation by 98% (Figure 1A, middle dot plot, Table S2). Among the five candidate compounds, cucurbitacin B, tripterin, sanguinarine, and valepotriate induced obvious cell death, which was not further investigated in this study. Further experiments indicated that physalin F inhibited SeV-triggered activation of ISRE in a dose-dependent manner, with the half maximal inhibitory concentration (IC_50_) at 1.0 μM (Figure 1A). RT-qPCR experiments indicated that physalin F dose-dependently inhibited SeV-induced transcription of downstream antiviral genes, including *IFNB1*, *IFI44*, and *CXCL10* in HEK293T cells and the human monocytes THP1 cells (Figure 1B). Phosphorylation of IRF3, STAT1, and STAT2 are hallmarks of virus-induced activation of the innate immune signaling pathways. Consistently, physalin F inhibited SeV-induced phosphorylation of IRF3 at Ser386 (IRF3^S386^), STAT1 at Y701 (STAT1^Y701^), and STAT2 at Y690 (STAT2^Y690^) at 8 h post-infection in HEK293T cells (Figure 1C, upper panels). In THP1 cells, physalin F inhibited SeV-induced phosphorylation of IRF3, STAT1, and STAT2 at 4 h post-infection, and had no marked effects on it at 8 h post-infection (Figure 1C, lower panels). These results suggest that physalin F negatively regulates the early onset of innate immune response to RNA virus. In contrast, physalin F had no marked effects on IFN-γ-induced transcription of the *IRF1* gene (Figure 1D) and phosphorylation of STAT1 (Figure 1E) in HEK293T cells. Collectively, these results suggest that physalin F inhibits SeV-induced innate immune response in human cell lines.

**Figure 2:**
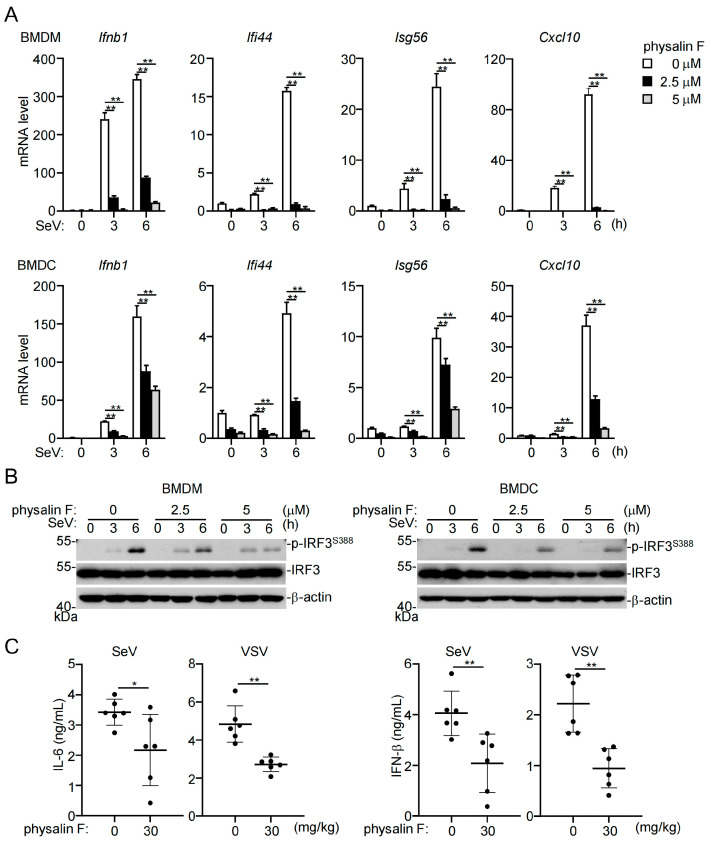
Physalin F suppresses RNA virus-induced innate immune response in primary mouse cells and mice. (**A**) Effects of physalin F on SeV-induced transcription of antiviral genes in mouse primary myeloid cells. BMDMs and BMDCs were treated with the indicated concentrations of physalin F for 0.5 h and then infected with SeV for the indicated times. The mRNA levels of the indicated downstream genes were measured by RT-qPCR. Data shown are mean ± SD; *n* = 3 technical replicates. *Gapdh* mRNA level was used as the internal control. (**B**) Effects of physalin F on SeV-induced phosphorylation of IRF3 in primary myeloid cells. BMDMs and BMDCs were treated as described in (**A**). Immunoblotting analysis was then performed with the indicated antibodies. Original Western blot images can be found in Figure S2A. (**C**) Effects of physalin F on RNA-virus induced IL 6 and IFN β production in the mouse sera. C57BL/6 mice (6-week-old female, *n* = 6 in each group) were treated by oral route with DMSO or physalin F (30 mg/kg) for 0.5 h, and then intraperitoneally injected with VSV or SeV (2 × 10^8^ pfu each mouse) for 8 h before measurement of serum cytokine levels by ELISA assay. Each data point represents an individual mouse. Data shown are mean ± SD; *n* = 6. The experiments in (**A**,**B**) were repeated at least two times with similar results. ns, not significant, * *p* < 0.05; ** *p* < 0.01.

We next investigated whether physalin F has similar functions in primary mouse cells and in mice. RT-qPCR experiments indicated that physalin F inhibited SeV-induced transcription of antiviral genes, including *Ifnb*, *Ifi44*, *Isg56*, and *Cxcl10*, in mouse bone marrow-derived macrophages (BMDMs) and dendritic cells (BMDCs) (Figure 2A). Consistently, physalin F inhibited SeV-induced phosphorylation of mouse IRF3^S388^ in BMDMs and BMDCs (Figure 2B). Additionally, oral administration of physalin F into mice inhibited SeV- and another RNA virus, vesicular stomatitis virus (VSV)-induced production of IL-6 and IFN-β in the sera of the infected mice (Figure 2C). These results suggest that physalin F inhibits RNA virus-triggered innate immune signaling in primary mouse immune cells and in mice.

### 3.2. Physalin F Directly Interacts with the Mitochondrial m-AAA Protease AFG3L2

**Figure 3:**
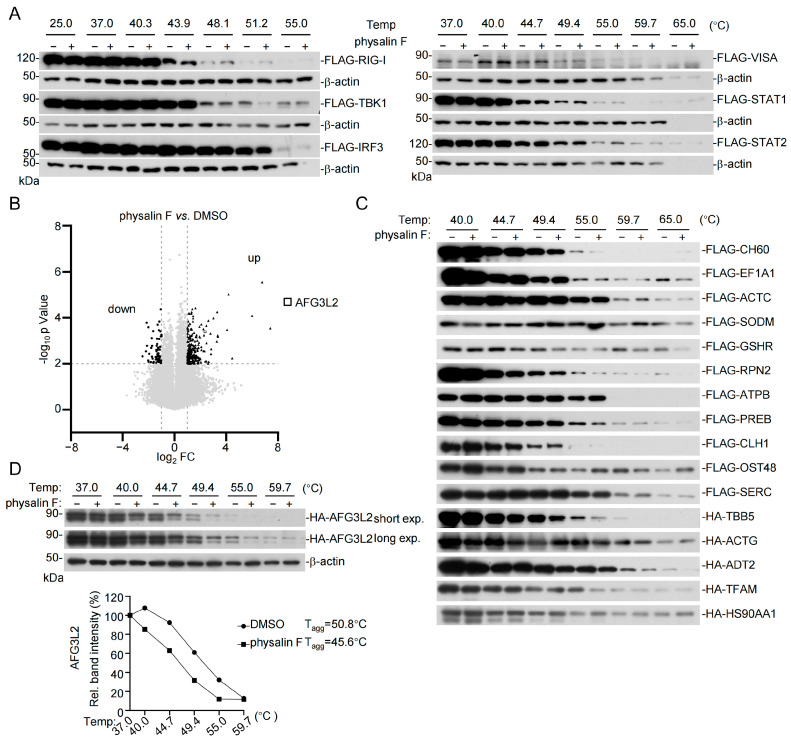
Identified AFG3L2 as the direct target of physalin F. (**A**) Effects of physalin F on the thermal stabilization of components of the RIG-I pathway. HEK293T cells were transfected with the indicated plasmids for 16 h, lysed with 1 mM phenylmethylsulfonyl fluoride in PBS, and then incubated with DMSO or physalin F (10 μM) for 2 h for CETSA. Soluble protein fractions at different temperatures were analyzed by immunoblots with the indicated antibodies. β-actin was used as a loading control. Original Western blot images can be found in Figure S3A. (**B**) Volcano plot depicting the different peptide abundance between DMSO-treated and physalin F-treated HEK293T cells in LiP-MS assay. Lysates from HEK293T cells were incubated with DMSO or physalin F (50 μM) for 10 min at 25 °C, followed by digestion with proteinase K for 10 min, and then analyzed by MS. Differentially expressed peptides were filtered based on *p* < 0.01 and a fold change > 2 and <−2. Solid triangles, upregulation; solid circles, downregulation; open squares, AFG3L2. (**C**) CETSA validation of the candidate physalin F targets identified by LiP-MS. HEK293T cells were transfected with the indicated plasmids for 16 h. The cell lysates were incubated with DMSO or AFG3L2 (10 μM) for 2 h before CETSA and immunoblotting analysis for the indicated proteins. Original Western blot images can be found in Figure S4A. (**D**) Physalin F decreases the thermal stability of AFG3L2. HEK293T cells were transfected with HA-AFG3L2 for 16 h. The cell lysates were incubated with DMSO or AFG3L2 (10 μM) for 2 h, followed by CETSA. The graphs show the quantification of AFG3L2 protein versus temperature points based on immunoblotting analysis. Soluble protein fractions at different temperatures were probed with the indicated antibodies, using β-actin as a loading control. Original Western blot images can be found in Figure S4B. The band intensities of AFG3L2 were normalized to β-actin and plotted as a percentage of the 37 °C control. A Boltzmann–sigmoidal curve was fit to estimate the T_agg_ for each treatment.

We next investigated whether physalin F directly targets signaling components in the RLR pathways by cellular thermal shift assay (CETSA) [46]. The results indicated that physalin F had no marked effects on the thermal stability of the examined components in the RLR pathway, including RIG-I, VISA, TBK1, IRF3, as well as STAT1, and STAT2 in the IFN-JAK-STAT pathway (Figure 3A), suggesting that these components in the RLR-mediated pathways are not direct targets of physalin F.

To unambiguously identify the targets of physalin F, we adopted the Limited Proteolysis coupled with Mass Spectrometry (LiP-MS) method [43]. Comparison of peptides derived from DMSO- or physalin F-treated HEK293T cells identified 319 significantly changed peptides mapping to 261 proteins, including 255 peptides that were up-regulated, and 64 peptides were down-regulated in physalin F-treated compared to mock-treated cells (Figure 3B). Then we performed a comprehensive scoring analysis, including the number of peptides, absolute log_2_FC, functional annotations (GO, KEGG, SL, Reactome, DO) counts, PPI degrees, special functional types, and ligand–target docking affinity, and identified 20 top physalin F targets (Table S3). Mammalian expression plasmids for 17 out of the top 20 candidates were successfully constructed and used for the CETSA to validate whether they are physalin F targets. The results indicated that physalin F induced thermal destabilization of AFG3L2 but not other examined candidate proteins (Figure 3C,D). The melt curve analysis indicated that upon binding with physalin F, the aggregation temperature (T_agg_) of AFG3L2 shifted from 50.8 °C to 45.6 °C (Figure 3D). These results suggest that physalin F binds to AFG3L2.

### 3.3. Inhibition of Innate Antiviral Signaling by Physalin F Is Mediated by AFG3L2

**Figure 4:**
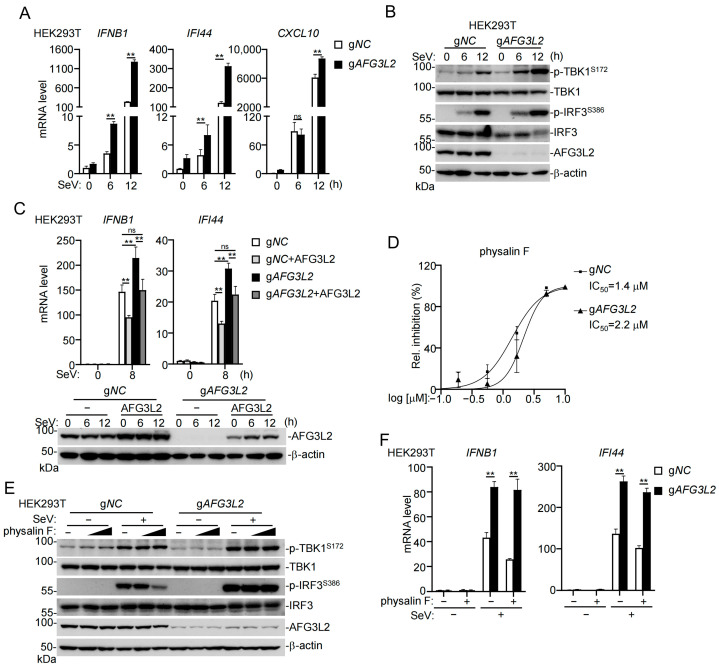
Physalin F negatively regulates innate antiviral response by targeting AFG3L2. (**A**) Deficiency of AFG3L2 enhances SeV-induced transcription of antiviral genes. Control or AFG3L2-deficient HEK293T cells (1 × 10^6^) were infected with SeV for the indicated times before RT-qPCR analysis of mRNA levels of the indicated antiviral effector genes. *GAPDH* mRNA level was used as the internal control. (**B**) Deficiency of AFG3L2 increases SeV-induced phosphorylation of TBK1 and IRF3. Control or AFG3L2-deficient HEK293T cells (1 × 10^6^) were infected with SeV for the indicated times before immunoblotting analysis with the indicated antibodies. Original Western blot images can be found in Figure S5A. (**C**) Effects of AFG3L2 on SeV-induced the transcription of antiviral genes. Control or AFG3L2-deficient HEK293T cells (1 × 10^6^) were reconstituted with AFG3L2 and then infected with SeV for 8 h before RT-qPCR analysis of mRNA levels of the indicated antiviral genes. *GAPDH* mRNA level was used as the internal control. Expression of AFG3L2 in the cells were measured by immunoblots. Original Western blot images can be found in Figure S5B. (**D**) IC_50_ for physalin F-mediated inhibition of SeV-induced ISRE activity. Control or AFG3L2-deficient HEK293T cells (5 × 10^4^) were transfected with an ISRE reporter plasmid (25 ng) for 16 h, and then treated with the indicated concentrations of physalin F for 0.5 h, followed by infection with SeV for 8 h before luciferase assays. The IC_50_ value was calculated from the dose–response curve. IC_50_ of physalin F in control cells was calculated to be 1.4 μM (95% CI: 1.2 to 1.6 μM); IC_50_ of physalin F in AFG3L2-deficient cells was calculated to be 2.2 μM (95% CI: 1.8 to 2.6 μM). (**E**) Effects of physalin F on SeV-induced phosphorylation of IRF3 in AFG3L2 knockdown cells. Control or AFG3L2-deficient HEK293T cells (1 × 10^6^) were treated with increased doses of physalin F (0, 1.25, 2.5 μM) for 0.5 h and then infected with SeV for 8 h. Immunoblotting analysis with the indicated antibodies was performed. Original Western blot images can be found in Figure S5C. (**F**) Effects of physalin F on SeV-induced transcription of antiviral genes in AFG3L2-deficient cells. Control or AFG3L2-deficient HEK293T cells (1 × 10^6^) were treated with physalin F (2.5 μM) for 0.5 h and then infected with SeV for 8 h before RT-qPCR analysis of mRNA levels of the indicated antiviral genes. *GAPDH* mRNA level was used as the internal control. The data in (**A**,**C**,**F**) are presented as mean ± SD, *n* = 3 technical replicates. ns, not significant, ** *p* < 0.01. All experiments were repeated at least two times with similar results.

We next investigated whether AFG3L2 mediates inhibition of innate antiviral signaling by physalin F. As shown in Figure 4A, knockdown of AFG3L2 promoted the transcription of *IFNB1*, *IFI44*, and *CXCL10* genes induced by SeV in HEK293T cells (Figure 4A). Consistently, SeV-induced phosphorylation of TBK1 and IRF3 was markedly enhanced in AFG3L2-deficient HEK293T cells (Figure 4B). In addition, overexpression of AFG3L2 inhibited SeV-induced transcription of *IFNB1* and *IFI44* in HEK293T cells, and reconstitution of AFG3L2 in AFG3L2-deficient cells reversed the increased transcription of downstream antiviral genes in AFG3L2-deficient cells upon SeV infection (Figure 4C). We further confirmed the dependence of AFG3L2 for physalin F-induced inhibition of innate antiviral response by reporter assays. We treated control and AFG3L2-deficient HEK293T cells transfected with an ISRE reporter plasmid with different doses of physalin F, and then infected with SeV for 8 h. Reporter assays indicated that physalin F inhibited SeV-induced ISRE activation at an IC_50_ of 1.4 μM in control cells, which was significantly lower than 2.2 μM in AFG3L2-deficient cells (Figure 4D). Additionally, physalin F inhibited SeV-induced phosphorylation of IRF3 and transcription of downstream *IFNB1* and *IFI44* genes in control but not AFG3L2-deficient cells (Figure 4E,F). Collectively, these results suggest that physalin F targets AFG3L2 to inhibit SeV-triggered innate antiviral signaling.

### 3.4. AFG3L2 Mediates Degradation of VISA

**Figure 5:**
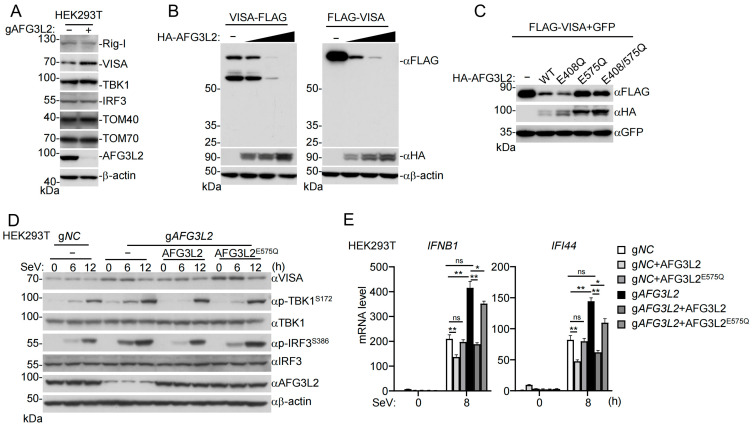
AFG3L2 negatively regulates antiviral innate immunity by degrading VISA. (**A**) Effects of AFG3L2 deficiency on endogenous levels of components in the RLR pathways. Control or AFG3L2-deficient HEK293T cells were collected for immunoblotting analysis with the indicated antibodies. Original Western blot images can be found in Figure S6A. (**B**) AFG3L2 promotes the degradation of VISA in a dose-dependent manner. HEK293T cells (1 × 10^6^) were transfected with increased amounts (0, 0.1, 0.3, 0.9 μg) of HA-AFG3L2 plasmid and C-terminal (VISA-FLAG) or N-terminal (FLAG-VISA) FLAG-tagged VISA for 24 h before immunoblotting analysis with the indicated antibodies. Original Western blot images can be found in Figure S6B. (**C**) Effects of wild-type AFG3L2 and its mutants on VISA degradation. HEK293T cells (1 × 10^6^) were transfected with the indicated plasmids for 24 h before immunoblotting analysis with the indicated antibodies. Original Western blot images can be found in Figure S6C. (**D**,**E**) Effects of wild-type AFG3L2 and its mutant on SeV-induced antiviral signaling. AFG3L2-deficient HEK293T cells (1 × 10^6^) reconstituted with wild-type AFG3L2 or AFG3L2^E575Q^ mutant were infected with SeV for the indicated times before immunoblotting analysis with the indicated antibodies (**D**) and RT-qPCR analysis of mRNA levels of the indicated antiviral genes (**E**). *GAPDH* mRNA level was used as the internal control. Original Western blot images in (D) can be found in Figure S6D. The data in (**E**) are presented as mean ± SD; *n* = 3 technical replicates. ns, not significant, * *p* < 0.05; ** *p* < 0.01. All experiments were repeated at least two times with similar results.

We next investigated the mechanisms responsible for AFG3L2-mediated inhibition of innate antiviral signaling. Since AFG3L2 is a mitochondrial *m*-AAA protease, we first investigated whether AFG3L2 down-regulates components of the RLR pathways. As shown in Figure 5A, knockdown of AFG3L2 in HEK293T cells resulted in a marked increase in endogenous VISA but not RIG-I, TBK1, or IRF3 levels (Figure 5A). In these experiments, knockdown of AFG3L2 in HEK293T cells had no marked effects on TOM40 and TOM70, which are also mitochondrial outer membrane proteins (Figure 5A). Overexpression of AFG3L2 caused down-regulation of either an N-terminal or C-terminal FLAG-tagged VISA in a dose-dependent manner (Figure 5B). The absence of detectable cleaved fragments of VISA in these experiments suggests that AFG3L2 promotes the proteolytic degradation rather than the limited cleavage of VISA (Figure 5B).

Previously, it has been demonstrated that E408 and E575 residues of AFG3L2 are critically important for its ATPase and protease activities, respectively [47,48,49]. To investigate whether the ATPase or the protease activity of AFG3L2 is essential for VISA degradation, we constructed the ATPase-defective mutant AFG3L2^E408Q^, the proteolytic inactive mutant AFG3L2^E575Q^, and the double mutant AFG3L2^E408/575Q^. Immunoblotting analysis indicated that overexpression of wild-type AFG3L2 and AFG3L2^E408Q^ but not AFG3L2^E575Q^ or AFG3L2^E408/575Q^ caused VISA degradation (Figure 5C), suggesting that the protease but not the ATPase activity of AFG3L2 is essential for regulating the endogenous level of VISA. Consistently, comparing to AFG3L2-deficient cells, cells reconstituted with wild-type AFG3L2 but not the protease-inactive AFG3L2^E575Q^ mutant down-regulated VISA level in uninfected and SeV-infected cells, and inhibited SeV-induced phosphorylation of TBK1^S172^ and IRF3^S386^ at 6 h post-infection (Figure 5D). In these experiments, reconstitution of AFG3L2 had no marked effects on VISA level and SeV-induced phosphorylation of TBK1^S172^ and IRF3^S386^ at 12 h post-infection. The simplest explanation for this observation is that AFG3L2 does not proteolytically degrade activated VISA, which has been shown to form large insoluble polymers/aggregates upon viral infection [10]. Additionally, compared to AFG3L2-deficient cells, cells reconstituted with wild-type AFG3L2 but not the protease-inactive AFG3L2^E575Q^ mutant inhibited SeV-induced transcription of *IFNB1* and *IFI44* genes at 8 h post-infection (Figure 5E). These results suggest that AFG3L2 negatively regulates innate antiviral signaling by promoting the proteolytic degradation of VISA under physiologic conditions.

### 3.5. Physalin F Interacts with AFG3L2 to Enhance Its Proteolytic Activity

**Figure 6:**
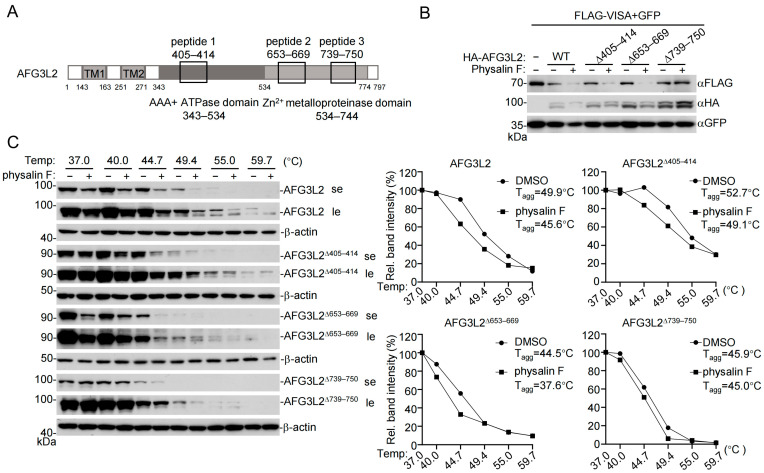
Mapping of the physalin F sites in AFG3L2. (**A**) A schematic presentation of AFG3L2 structures. The peptides identified by LIP MS are indicated, which are located in the ATPase domain and zinc metalloproteinase domain. (**B**) Effects of physalin F on the peptidase activity of AFG3L2. HEK293T cells were transfected with the indicated plasmids for 16 h and treated with DMSO or physalin F (10 μM) for 2 h before immunoblotting analysis with the indicated antibodies. se, short exposure; le, long exposure. Original Western blot images can be found in Figure S7A. (**C**) CETSA analysis of physalin F binding to wild-type AFG3L2 and its truncations. HEK293T cells were transfected with the indicated plasmids for 16 h. The cell lysates were incubated with DMSO or AFG3L2 (10 μM) for 2 h, and followed by CETSA. Soluble protein fractions at different temperatures were analyzed by immunoblots with the indicated antibodies. Original Western blot images can be found in Figure S7B. The band intensities of AFG3L2 were normalized to β-actin and plotted as a percentage of the 37 °C control. A Boltzmann sigmoidal curve was fit to estimate the T_agg_ for each treatment. All experiments were repeated at least two times with similar results.

In our earlier LiP-MS assays, three peptides in AFG3L2 were identified to be protected from broad-spectrum proteolytic digestion by physalin F, including (1) FIDEIDAVGR, aa405–414, located in the Walker B motif of the AAA+ ATPase domain; (2) VTQSAYAQIVQFGMNEK, aa653–669; and (3) NDMVELLGPRPF, aa739–750, located within the zinc metalloproteinase domain (Figure 6A). To identify physalin F-binding sites on AFG3L2, we generated three AFG3L2 truncations, including AFG3L2^Δ405–441^, AFG3L2^Δ659–669^, and AFG3L2^Δ739–750^, and examined the effects of physalin F on VISA degradation mediated by these mutants. The results indicated that physalin F promoted VISA degradation by wild-type AFG3L2, AFG3L2^Δ405–441^ and AFG3L2^Δ659–669^ but not AFG3L2^Δ739–750^. These results suggest that aa739–750 of AFG3L2 is required for physalin F to enhance its proteolytic degradation of VISA (Figure 6B). CETSA revealed that physalin F treatment induced the thermal shift in wild-type AFG3L2, AFG3L2^Δ405–441^ and AFG3L2^Δ659–669^ but not AFG3L2^Δ739–750^ (Figure 6C). These results suggest that physalin F directly binds to aa739–750 of AFG3L2 to promote its proteolytic degradation of VISA.

## 4. Discussion

Sensing of microbial components by PRR is essential for effective host defense, whereas abnormal activation of PRR-triggered innate immune response leads to a variety of immunopathology [22,50,51,52]. In this study, we identified the natural molecule physalin F as an inhibitor of RNA virus-induced innate immune response. We further discovered that physalin F targets the mitochondrial protease AFG3L2, promotes its proteolytic activity, and degrades VISA, a central adaptor protein in the RLR-triggered innate immune pathways. Therefore, our studies reveal a novel negative regulatory mechanism on innate immune response to RNA virus.

Our experiments suggest that physalin F inhibits innate immune response to RNA virus in both human and mouse cells, as well as in mice. Utilizing LiP-MS, we identified AFG3L2 as the direct target of physalin F. Several lines of evidence suggest physalin F inhibits innate antiviral response by targeting AFG3L2. Firstly, CETSA indicated that binding of physalin F resulted in a negative thermal shift in the melting curve of AFG3L2. Second, knockdown of AFG3L2 potentiated SeV-triggered transcription of downstream antiviral genes and the phosphorylation of IRF3, while overexpression of AFG3L2 had opposite effects. Consistently, AFG3L2-deficiency conferred resistance to the inhibitory effects of physalin F on SeV-triggered induction of downstream antiviral genes.

**Figure 7:**
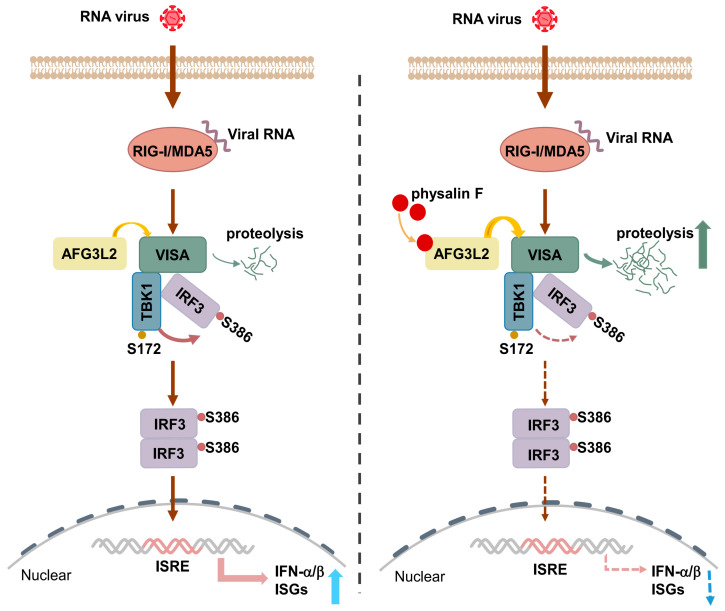
A work model for suppression of innate immune response to RNA virus by physalin F-AFG3L2. The mitochondrial AAA protease AFG3L2 constitutively promotes degradation of VISA/MAVS, a central adaptor in RLR-triggered innate antiviral signaling pathways, under physiological conditions. This maintains VISA at a proper level at which auto-activation of VISA-mediated innate immune response/inflammation is prevented in uninfected cells, while efficient innate immune response is initiated upon infection of RNA virus. The natural molecule compound physalin F binds to AFG3L2, promotes its proteolytic activity and degradation of VISA, and thereby suppresses the innate immune response to RNA virus.

Our experiments suggest that physalin F increases AFG3L2 proteolytic activity, which promotes the degradation of VISA to inhibit innate antiviral response (Figure 7). Knockdown of AFG3L2 increased the abundance of VISA under physiological conditions. Reconstitution of AFG3L2 cells with wild-type AFG3L2 but not the protease-inactive mutant AFG3L2^E575Q^ decreased the level of VISA and SeV-induced phosphorylation of IRF3, whereas physalin F enhanced the degradation of VISA mediated by overexpression of wild-type AFG3L2 but not AFG3L2^Δ739–750^, the mutant in which its physalin F-binding site was deleted. Collectively, these findings suggest that physalin F engages aa739–750 of AFG3L2 to promote its protease activity and VISA degradation, thereby negatively regulating RLR-mediating antiviral immune response. Although we have mapped the binding interface of physalin F to aa739–750 of AFG3L2, the precise residues remain to be identified. It also remains to be elucidated whether the binding of physalin F to AFG3L2 induces its conformational changes to promote its enzymatic activity. AFG3L2 is known to proteolytically cleave substrates into small peptides rather than generating stable cleavage intermediates with a preference for hydrophobic residues at the P1′ cleavage site [48,49]. This may explain our observations that AFG3L2 causes degradation of full-length VISA without detectable processed fragments. Since it is technically difficult to prepare recombinant AFG3L2 due to its property as a large (797aa) transmembrane protein, our study did not offer direct evidence by in vitro assays to demonstrate that VISA is a bona fide substrate of AFG3L2.

Physalin F has been reported to exhibit anti-tumor and anti-inflammatory activities [53,54]. Physalin F induces the accumulation of ROS and activation of caspase-3, leading to apoptosis of various cancer cell lines [34,35]. It has also been demonstrated that physalin F inhibits the activation of NF-κB in renal cancer cell lines and impairs the Wnt/β-catenin pathway by accelerating β-catenin degradation [35,55]. Additionally, it has been shown that physalin F causes a concentration-dependent reduction in lymphocytes in HTLV-1-infected patients [33], and inhibits the secretion of cytokines such as IL-2, IL-4, IL-10, and IFN-γ [31,56]. Physalin F inhibits NO production and inducible nitric oxide synthase (iNOS) expression in activated macrophages [57]. Physalin F has been demonstrated efficacy superior to dexamethasone in suppressing LPS induced TNFα and IL-6 production, thereby protecting mice against a lethal endotoxic shock and reducing inflammation in allograft transplantation models [57]. The direct molecular target of physalin F that mediates its above mentioned effects are undefined. Although we identified AFG3L2 as a target of physalin F, additional targets of physalin F may exist because of the limitations of the LiP-MS method used in our current study. Whether AFG3L2 and unidentified targets are involved in the effects of physalin F on these divergent cellular processes needs to be investigated in future studies.

AFG3L2 is a critical subunit of the mitochondrial *m*-AAA protease, involved in maintaining mitochondrial proteostasis by recognizing and clearing misfolded proteins [58]. Previously, it has been reported that in model organisms, including mice and *Drosophila*, AFG3L2 is essential for axon development, cell survival, and the mitochondrial function integrity [59,60,61,62]. Evidence from yeast demonstrates that AFG3L2 mediates the proteolytic processing of the ribosomal protein MrpL32, a key component required for mitochondrial ribosome assembly [63]. Loss of AFG3L2 activates the protease OMA1, which cleaves long-form OPA1 (L-OPA1), thereby inhibiting mitochondrial fusion and promoting mitochondrial network fragmentation [64]. AFG3L2 also degrades essential mitochondrial calcium uniporter (MCU) regulator element (EMRE) to prevent mitochondrial calcium overload and the opening of the mitochondrial permeability transition pore (mPTP) [65]. Notably, dysregulation of mitochondrial calcium homeostasis, leading to cellular apoptosis, while mPTP opening induces mtDNA leakage and the subsequent activation of the cytosolic cGAS-MITA/STING innate immune pathway [66]. It has also been shown that mitochondrial inner membrane (IMM) GSH transporter SLC25A39 is rapidly degraded by AFG3L2 under basal conditions, and knockout of AFG3L2 does not affect the mitochondrial outer membrane protein VDAC [67]. Reduced AFG3L2 expression in lung adenocarcinoma stabilizes SLC25A39 and enhances oxidative phosphorylation (OXPHOS) and ROS handling, thereby promoting tumor progression [68]. These studies suggest that AFG3L2 is involved in mitochondrial homeostasis and various cellular processes.

Our current study establishes that AFG3L2 regulates VISA level under physiological conditions, therefore adding to the biological processes regulated by AFG3L2. Previously, it has been demonstrated that VISA is an outer mitochondrial membrane (OMM) protein [7,8], while AFG3L2 is embedded in IMM [58]. This raises an outstanding question on whether and how AFG3L2 directly regulates the VISA level. Although IMM and OMM exist as independent components, they are physically and functionally interconnected by the mitochondrial contact site and cristae organizing system (MICOS) complex, which localizes to the IMM and regulates the formation and communication between the IMM and OMM [69]. In this context, a previous study has demonstrated that Yme1, an IMM protease, degrades OMM proteins TOM22 and OM45 [70]. It is also possible that AFG3L2 and VISA can dynamically co-localize on both IMM and OMM, thereby enabling direct proteolytic degradation of VISA by AFG3L2. Additionally, AFG3L2 may target other components to regulate the homeostasis of mitochondria, thereby indirectly regulating the VISA level.

While our study identifies physalin F as a potential inhibitor of RNA virus-induced type I IFN production, and points to its potential as a therapeutic agent for excessive innate immune/inflammatory responses, the clinical application of physalin F requires careful context-dependent consideration and additional preclinical studies. Nevertheless, our study suggests that targeting AFG3L2, such as by physalin F, may provide potential therapeutic benefits for RNA virus-induced infectious and inflammatory diseases.

## 5. Conclusions

In conclusion, our studies suggest that AFG3L2 promotes VISA degradation under physiological conditions. This may contribute to a dynamic equilibrium of VISA level that is important for preventing innate immune activity/autoinflammation under physiological conditions while efficiently initiating innate immune response upon viral infection. Therefore, our current study reveals a previously unidentified negative regulatory mechanism of RLR-triggered innate immune response to RNA virus. Additionally, we show that the natural compound physalin F promotes AFG3L2 proteolytic activity to regulate the degradation of VISA and suppress innate antiviral response, offering a promising therapeutic approach to disorders associated with infection of the RNA virus and AFG3L2 dysfunctions.

## Data Availability

The original contributions presented in this study are included in the article/Supplementary Material. Further inquiries can be directed to the corresponding authors.

## Supplementary Materials

The following supporting information can be downloaded at: https://www.mdpi.com/article/10.3390/pathogens15010074/s1, Table S1. Effects of the sub-pools of 903 compounds on SeV-induced ISRE activation; Table S2. Effects of 48 compounds on SeV-induced ISRE activity; Table S3: A list of candidate proteins targeted by physalin F from LiP-MS. Figure S1: Original western blot images for Figure 1C,E; Figure S2: Original western blot images for Figure 2B; Figure S3: Original western blot images for Figure 3A; Figure S4: Original western blot images for Figure 3C,D; Figure S5: Original western blot images for Figure 4B,C,E; Figure S6: Original western blot images for Figure 5A–D; Figure S7: Original western blot images for Figure 6B,C.

## Abbreviations

The following abbreviations are used in this manuscript:

AFG	ATPase family gene
AFG3L2	AFG3-like matrix AAA peptidase subunit 2
AGS	Aicardi Goutieres syndrome
BMDCs	bone marrow-derived dendritic cells
BMDMs	bone marrow-derived macrophages
CARD	caspase activation and recruitment domain
CETSA	cellular thermal shift assay
CRISPR	clustered regularly interspaced short palindromic repeats
DMSO	dimethyl sulfoxide
EGCG	epigallocatechin gallate
EMRE	essential MCU regulator element
GPATCH3	G patch domain containing protein 3
HEK293T	human embryonic kidney
HTLV-1	human T lymphotropic virus type 1
IC_50_	half-maximal inhibitory concentration
IFNs	type I interferons
IRF3	interferon regulatory transcription factor 3
IMM	inner mitochondrial membrane
ISRE	IFN-stimulated response element
LiP-MS	Limited Proteolysis coupled with Mass Spectrometry
LPS	Lipopolysaccharide
*m*-AAA	matrix ATPase associated with diverse cellular activities
MDA5	melanoma differentiation-associated gene 5
MICOS	mitochondrial contact site and cristae organizing system
mPTP	mitochondrial permeability transition pore
MUC	mitochondrial calcium uniporter
OMM	outer mitochondrial membrane
OXPHOS	oxidative phosphorylation
RIG-I	retinoic acid-inducible gene I
RLRs	RIG I like receptors
ROS	reactive oxygen species
PRRs	pattern recognition receptors
SeV	Sendai virus
SLE	Systemic lupus erythematosus
SNX8	Several proteins, including sorting nexin 8
T_agg_	aggregation temperature
TBK1	TANK-binding kinase 1
THP1	human monocytes
TNFα	tumor necrosis factor alpha
TRX2	Thioredoxin 2
VISA	virus-induced signaling adaptor
VSV	Vesicular stomatitis virus
