# Supplementary material for: Physalin F Promotes AFG3L2-Mediated Degradation of VISA/MAVS to Suppress Innate Immune Response to RNA Virus
Source: Pathogens. 2026 Jan 9;15(1):74. doi: 10.3390/pathogens15010074 (PMC12844614; doi:10.3390/pathogens15010074)
Supplement: Supplementary file 1 [file pathogens-15-00074-s001.zip › pathogens-4090213-supplementary.pdf]

Table S1 Effects of the sub-pools of 903 compounds on SeV-induced ISRE activation

	Sub-pools				Rel. activity of ISRE (log_{10} %)
1	Monobutyl Phthalate	Saccharin	Zoledronic Acid	Norharmane	1.83
2	Phthalic acid mono-2-ethyl hexyl ester	Oxytetracycline	PHYTOL	Herniarin	1.84
3	Cedryl acetate	Adenosine 5'-monophosph ate monohydrate	(S)-(+)-Carvon e	alpha-Tocophe rolquinone	1.92
4	Triacetin	Esculetin	Vanillin	β-Caryophylle ne	1.92
5	Ketoisophoro ne	Phloretin	Perillyl alcohol	Maltopentaose	1.82
6	Retinyl acetate	Piperine	Angelic Acid	Steviol	2.00
7	Trans-Aneth ole	Pinocembrin	Liquiritigenin	N4-Acetylcytid ine	1.93
8	Crotamiton	Naringenin	Ursonic Acid	2,6-Dimethoxy quinone	1.82
9	Phytic acid	18α-Glycyrrheti nic acid	Eupatilin	N-Nitroso-N-methylurea	1.87
10	Citicoline	Madecassic acid	Rebaudioside C	steviolbioside	1.89
11	Menadione	Limonin	Gypenoside XVII	2'-Deoxyuridin e 5'-monophosp hate disodium	1.96
12	Ellagic acid	Neohesperidin	Licochalcone A	Indole-3-butyri c acid	1.92
13	Promethazin e	Betulinic acid	Obacunone	Lumichrome	1.92
14	Edaravone	Isoalantolactone	Hydroxytyros ol	Iprodione	1.83
15	6-Mercaptop urine	Cryptotanshino ne	Protopine	Cyclothiazide	1.83
16	Urethane	Rutin	20(S)-Ginsenos ide Rg3	Diflubenzuron	1.92
17	Cefpiramide acid	Catechin	Amentoflavon e	GUANOSINE 3':5'-CYCLIC MONOPHOSP HATE SOD	1.93
18	Escitalopram	Ethylparaben	Carbocisteine	Juglone	1.84
19	Fluticasone (propionate)	Cyromazine	Polydatin	Oxytocin	1.89
20	Valdecoxib	Propafenone	Aucubin	Pipobroman	1.90
21	N-Acetylproc ainamide	Desloratadine	Ginsenoside F1	Glycochenode oxycholic Acid	1.85

22	Amcinonide	Cyclamic acid	Cucurbitacin B	Gonadorelin Acetate (33515-09-2 free base)	0.28
23	Hexylresorcinol	Butylparaben	Zingerone	Gynostemma Extract	1.94
24	10-Undecenoic acid	L-Ascorbic acid	Curdione	Indole-3-pyruvic acid	1.98
25	Arbutin	Sodium butanoate	Curcumenol	Rhamnose	1.99
26	Ethylvanillin	Sucralose	Galangin	Sodium phenylpyruvate	1.93
27	Mequinol	(-)-Menthol	Hydroxysafflor yellow A	Eicosapentaenoic Acid	1.94
28	Methyldopa	Rapamycin	Vitamin D3	TBHQ	1.27
29	Esculin	Iohexol	alpha-Asarone	Docosahexaenoic Acid	1.87
30	Vanillin acetate	Umbelliferone	Methyl Paraben	Arachidonic acid	1.92
31	Dihydrocaffeic acid	Diosmetin	Pseudoginsenoside F11	3-Methyl-2-oxobutanoic acid	1.83
32	Isohomovanillic acid	5-Amino-3H-imidazole-4-Carboxamide	Ginsenoside F2	Dodecanedioic acid diammonium salt	2.05
33	7-Methylxanthine	Chitosan (MW 150000)	Tetrahydrocurcumin	Actriol	2.12
34	Myricetin	Aloe emodin	Tracheloside	Tomatine	1.78
35	Glycyrrhizic acid	Indole-3-carbinol	4-Hydroxybenzyl alcohol	[10]-Shogaol	1.92
36	Tyrosol	Desvenlafaxine	Maslinic acid	N-(3-Phenylpropionyl)glycine	1.96
37	Syringic acid	Silibinin	Esculentoside A	Theaflavin 3,3'-digallate	1.92
38	Maltitol	Etonogestrel	3-(2,4-Dihydroxyphenyl)propanoic acid	Benzamide	1.95
39	Lactose	Allylestrenol	Diosgenin glucoside	Taurolithocholic acid sodium salt	2.05
40	Adipic acid	Kaempferol	Jujuboside B	Hemin	2.06
41	Daidzein	Trigonelline	Oleuropein	Epibrassinolide	2.03
42	Irinotecan Hydrochloride	Notoginsenoside R1	Tectorigenin	MALTOTETRAOSE	2.01
43	5-Fluorouracil	Ginsenoside Rg1	Ginsenoside Ro	zerumbone	2.11

44	Tylosin	Alantolactone	Euphol	Ginsenoside Rb2	1.78
45	Amoxicillin	Stevioside	3',4'-Dihydroxyacetophenone	6-Methylcoumarin	2.13
46	Docetaxel	Costunolide	N6-methyladenosine	Maltol	1.61
47	Hesperidin	Morin	Dihydrocapsaicin	Paradol	1.84
48	Imazalil	Ginsenoside Rb1	ML240	CAFESTOL	2.03
49	Fenbendazole	Ginsenoside Re	2,6-Dimethoxybenzoic acid	7,4'-Di-O-methylapigenin	1.70
50	Norfloxacin	Isorhamnetin	Calcifediol	Stachydrine	1.96
51	Cinchonidine	Crustecdysone	Calcitriol	Veratraldehyde	1.85
52	Nonivamide	Ginsenoside Rd	(-)-(S)-Equol	Guggulsterone	1.98
53	Tetracycline	Arctigenin	Palmitoylethanolamide	cis,cis-Muconic acid	1.48
54	Dicloralurea	(-)-Epicatechin	1-Octacosanol	H-Gly-Pro-OH	2.01
55	Cytarabine	Nobiletin	p-Anisic acid	β-Apo-8'-carotenal	1.93
56	Neohesperidin Dihydrochalcone	Asiaticoside	Tussilagone	1-Kestose	1.96
57	Gibberellic acid	Ginsenoside Rh2	Cinnamyl alcohol	1,3-Dicaffeoylquinic acid	1.87
58	Caryophyllene oxide	Genipin	Ginkgolide J	Menthol	1.88
59	β-Cyclodextrin	Liquiritin	Methyl palmitate	Oglufanide	1.91
60	1,3-Diphenylurea	Meglutol	Methyl stearate	Pyrroloquinoline quinone	1.90
61	Phloracetophenone	Ginsenoside Rg2	Methyl Linoleate	MALTOHEXAOSE	1.99
62	Xanthoxylin	Betulin	2'-Hydroxyacetophenone	Nerol	1.92
63	Asaraldehyde	Carbendazim	Vanillic Acid	Rebaudioside B	1.97
64	4-Hydroxychalcone	6-Thioguanine	Triolein	Propylparaben	1.96
65	D-(+)-Melezitose hydrate	Isocorydine	Asiaticoside B	TriacetonaMine	1.97
66	Geniposidic Acid	Sanguinarine	Didymin	Tiglic acid	0.43
67	Sclareol	Ginsenoside Rh1	Oxalic acid	2-Furoic acid	1.83
68	Piceatannol	Ginkgolide A	3,4-Dicaffeoylquinic acid	Levulinic acid	1.87
69	Nicotinamide N-oxide	Ginkgolide C	Xanthosine	Tricarballylic acid	1.99

70	5-Aminosalicylic Acid	Ginkgolide B	trans-Zeatin	Methyl nicotinate	1.99
71	Ursolic acid	Tangeretin	Dehydroabietic acid	Undecanedioic acid	1.89
72	Natamycin	Paliperidone	L-Gulono-1,4-lactone	Ginsenoside Rh4	1.96
73	Quinine	Mycophenolic acid	Diosbulbin B	2-(4-Methoxyphenyl)acetic acid	1.87
74	2-Naphthol	Madecassoside	Xanthotoxol	Acetylpyrazine	1.99
75	Troxerutin	Bilobalide	Damascenone	Turanose	2.04
76	Carbadox	DL-O-Tyrosine	(E)-Ethyl p-methoxycinnamate	5a-Pregnane-3,20-dione	1.88
77	Ascorbyl palmitate	Harmine	Isomangiferin	2-Hydroxy-2-methylbutanoic acid	1.70
78	Dinitolmide	Levobupivacaine	Acevaltrate	Fenchyl Alcohol	1.49
79	Gallic acid	Guaiazulene	Apiin	Diethyl malonate	1.95
80	Diosmin	Rebaudioside A	Transcrocetin	Benzyl acetate	1.98
81	Phthalic acid	2-Methoxy-4-vinylphenol	Miquelianin	Angelicain	1.96
82	Isophorone	2-Methyl-4-pentenoic Acid	CHLOROQUINE	Juglanin	1.94
83	Octyl acetate	5α-Cholestane	β-Elemene	D-Desthiobiotin	2.00
84	Musk ketone	N-Acetyl-L-methionine	5,7,4'-Trimethoxyflavone	D-Ribonolactone	2.09
85	Geraniol	2-Methylheptanoic Acid	Hydroxy-α-sanshool	3-Aminobutanoic acid	2.00
86	Cysteamine hydrochloride	α-Angelica lactone	Pseudouridine	ATP	2.09
87	Isovaleric acid	Aminopterin	3-Methoxyphenylacetic acid	Lactitol	2.12
88	Hexyl hexanoate	16-Dehydroprogesterone	Coumaran	Butyl isothiocyanate	2.14
89	cis-3-Hexenyl hexanoate	O-Acetylserine	1,3,7-Trimethyluric acid	Soyasapogenol B	2.02
90	Geranyl acetate	Nudifloramide	1-Methyladenosine	3-Acetyl-beta-boswellic acid	2.14
91	1-Undecanol	Sulbactum Sodium	1-Oleoyl-sn-glycero-3-phosphocholine	Oleoside 11-methyl ester	1.94
92	Docosanoic acid	Glucovanillin	LysoPC(14:0/0:0)	PROPYL DISULFIDE	1.96

93	Delta-Tocopherol	medicagenic acid	physalin F	1-Eicosanol	1.08
94	Cuminaldehyde	Prosapogenin A	4-Methylbiphenyl	Dimethyl sulfone	2.02
95	4-Ethylphenol	5'-Methylthioadenosine	p-Toluic Acid	Kahweol	1.89
96	JOSAMYCIN	4-Methyloctanoic acid	N-Acetyl-L-tryptophan	Tomatidine	2.05
97	Menaquinone-4	1-Furfurylpyrrole	8-Hydroxyguanosine	Dihydrolanosterol	2.05
98	(E)-m-Coumaric acid	2-Benzylsuccinic acid	β-Boswellic acid	Epirosmanol	2.04
99	5-Phenylvaleric Acid	γ-Hexalactone	Miltirone	Protirelin Acetate(24305-27-9 free base)	1.99
100	Ursocholic acid	Decyl aldehyde	Desmethylglycitein	Dihydromethysticin	1.96
101	2,6-Dihydroxybenzoic acid	cis-Jasmone	Piperlongumine	5-Geranoxy-7-methoxycoumarin	1.99
102	SDMA	Dihydrojasmone	4'-Methylacetophenone	Nordihydrocapsaicin	1.94
103	Flavokawain C	4-Pentenoic acid	4-Hydroxybenzyl cyanide	Serotonin	1.99
104	3,3-Dimethylglutaric acid	Methyl 2-furoate	Dihydrokaempferol	3'-Adenylic acid	1.98
105	4-Isopropylbenzyl Alcohol	Ethyl pyruvate	Helichrysetin	Eicosapentaenoic acid ethyl ester	1.93
106	2-Methoxybenzoic acid	2-Ethylbutyric Acid	Procyanidin B1	Menthyl isovalerate	2.00
107	3-Methoxybenzoic acid	Dimethyl Trisulfide	Loxapine	(-)-Hydroxycitric acid	1.97
108	Diethyl phosphate	N-Acetyl-L-arginine	Phenelzine sulfate	17a-Hydroxypregnenolone	1.92
109	Guanidinosuccinic acid	Acetamide	Mesaconic acid	Simmondsin	1.91
110	Rebaudioside D	α-Terpinene	Urolithin B	Grapiprant	1.93
111	Ambroxide	Linalyl Acetate	Ricinoleic acid	Withanolide A	1.90
112	Indole-3-carboxylic acid	2-Hydroxycinnamic acid	Fucoxanthin	Acetoacetic acid sodium salt	1.82
113	Glycolic acid	Trans-2-Hexenal	7-Methoxyrosmanol	1,2-Dipalmitoyl-sn-glycerol 3-phosphate sodium	1.79
114	5-Methyl-2'-deoxycytidine	Diethyl succinate	Harmalol	Luzindole	2.04

115	6-(Dimethylamino)purine	Indole-3-carboxaldehyde	γ-Glu-Phe TFA(7432-24-8 free base)	Isoguanine	1.98
116	3-Hydroxymandelic Acid	Epoxylinalool	Sitostenone	5-Methyltetrahydrofolic acid	2.06
117	Asymmetric dimethylarginine	1,2-dioleoyl-sn-glycero-3-phosphocholine	Oleoylethanolamide	3,4-Dihydroxymandelic acid	2.09
118	Guanosine 5'-diphosphate	Triacontanoic Acid	Spaglumic acid	Cichoriin	2.11
119	5-Hydroxytryptophol	d-Neomenthol	Adenosylcobalamin	Eleutheroside B1	2.06
120	6-Biopterin	1,3-Dimethyluric acid	Creosol	Sevoflurane	2.01
121	Alloepipregnanolone	Octacosanoic acid	Miglustat	Polyporusterone B	1.90
122	7-Methylguanosine	3-Amino-4-methylpentanoic acid	1,5-Anhydrosorbitol	O-Nornuciferine	1.94
123	5-Methylcytidine	3-Hydroxypicolinic acid	Endomorphin 1	3-Epioleanolic acid	2.03
124	2,3-Diaminopropionic acid	Nicotinamide riboside chloride	Asparagusic acid	Euscaphic acid	2.20
125	γ-L-Glutamyl-L-alanine	Tetradecanedioic acid	Mulberrin	Myrcene	2.12
126	Urolithin A	2-Hydroxycaprylic acid	Quercetagitrin	Pomolic acid	1.87
127	all-trans-4-Oxoretinoic acid	α-Thujone	1-Caffeoylquinic acid	Glabrone	2.02
128	Hexacosanoic acid	Cyclo(his-pro)	Secoisolariciresinol	2-Undecanol	1.99
129	(±) Anabasine	piperitone	Soyasaponin Ba	3'-Demethylnobiletin	1.97
130	2-Ketoglutaric acid	10Z-Nonadecenoic acid	Methyl anthranilate	Octadecanedioic acid	1.91
131	2,4-Dihydroxybenzoic acid	4,6-Dioxoheptanoic acid	Methyl anisate	trans-Vaccenic acid	1.94
132	4-Phenylbutyric acid	Pentacosanoic acid	2,4-D	Monomethyl fumarate	2.01
133	TRPM8 antagonist WS-3	Arborine	Glycogen, Mussel	Amitriptyline	1.97
134	Faltan	Glycyrrhetic acid 3-O-β-D-glucuronide	(-)-Isopulegol	Kukoamine A	1.86
135	Endomorphin 2	Glabrol	Adrenic Acid	Gentisin	1.93

136	Alendronic Acid	ATMP	Lipoic acid	(-)-Carvone	1.99
137	Nifursol	β-Pinene	7-Ketocholesterol	1-Octanol	2.04
138	Tricyclazole	Aloe-emodin-8-O-β-D-glucopyranoside	Dihydrodaidzein	nerolidol acetate	1.99
139	Pentadecanoic acid	Testosterone acetate	Tirucallol	Perseitol	2.02
140	Cyclohexane carboxylic Acid	SAH	Mecillinam	(-)-Menthone	1.94
141	Phenprocoumon	Linoleoyl Ethanolamide	Isopropyl myristate	Umbelliprenin	1.88
142	Farnesol	Cinacalcet	Phenyl salicylate	Gartanin	1.83
143	2-Methylhexanoic acid	Trifluoperazine	Ethyl salicylate	(-)-Bornyl acetate	1.83
144	(Z)-Aconitic acid	Abscisic Acid	UDP-g acid	1-Methylxanthine	1.89
145	Terpinen-4-ol	Diallyl Trisulfide	11-Keto-beta-boswellic acid	4-Hydroxynonenal	1.33
146	4-Methoxybenzaldehyde	Hexa-D-arginine	Docosapentaenoic acid 22n-3	D-Cysteine	1.99
147	Dibutyl sebacate	Nitecapone	N2,N2-Dimethylguanosine	cis-3-Hexen-1-ol	1.96
148	Sodium Thiocyanate	Cis-5-Dodecenoic Acid	3-Hydroxyglutaric acid	Anandamide	2.09
149	Terephthalic acid	Nicotinamide riboside	ETHYL OCTANOATE	D-Threitol	1.97
150	Sorbic acid	N-Acetylputrescine hydrochloride	Luteolinidin chloride	1,2-Dimyristoyl-sn-glycero-3-phosphocholine	1.91
151	Phenylglyoxylic acid	(S)-b-aminoisobutyric acid	γ-Tocotrienol	1,3-BUTANEDIOL	1.95
152	Citronellyl acetate	7,8-Dihydro-L-biopterin	2-Mercaptobenzothiazole	(S)-3-Hydroxybutanoic acid	1.98
153	Nonadecanoic acid	Coenzyme A	Nigakinone	11-Beta-hydroxyandrostenedione	1.97
154	o-Toluic acid	Bradykinin	Levomenol	L-Methionine sulfoxide	2.05
155	Citronellal	Neuromedin B	Pyropheophorbide-a	Methyl 3-phenylpropanoate	2.01
156	Isonicotinic acid	pyridostigmine	3,5-Diiodo-L-tyrosine dihydrate	Methyl myristate	1.81

157	3-Indoleacetonitrile	Substance P	8-Deoxygartanin	1-Hexanol	1.96
158	Saquinavir	N-Acetylcarnosine	DiosMetin 7-O-β-D-Glucuronide	Methyl octanoate	2.02
159	Rhodamine B	Sakuranetin	1,4-Cineole	PHYTOSPHINGOSINE	2.00
160	3-Hydroxycoumarin	Deoxycytidine triphosphate	Chrysoeriol	1-Pentadecanol	1.86
161	Methyl Laurate	γ-Cyclodextrin	Bicine	DMSO	2.04
162	Safranal	N,N,O-Tridesmethylvenlafaxine	Creatine monohydrate	Ferroheme	2.06
163	2'-FUCOSYL LACTOSE	N-Caffeoyltryptophan	Methyl cyclohexanecarboxylate	3-Methyl-L-histidine	2.08
164	7α-Hydroxycholesterol	Bestim	2,5-Dimethyl-3(2H)-furanone	Erythrodiol	2.07
165	Pellitorine	(-)-α-Pinene	4-Methylpentanoic acid	Solasodine	2.15
166	Homodihydrocapsaicin I	Octadecanal	3-Amino-2-oxazolidinone	Coenzyme Q9	2.08
167	1-Dodecanol	Brassinolide	3-Methylxanthine	Scyllo-Inositol	2.11
168	Ethyl oleate	Methyl dihydrojasmonate	Methylsyringol	DL-Dopa	2.14
169	Cyperotundone	Dimercaprol	(-)-Epigallocatechin Gallate	Anserine	2.01
170	Sanguisorbigenin	18β-Glycyrrhetinic acid	Curcumol	H-Val-Ala-OH	1.97
171	Prudomestin	Acetaminophen	β-Elemonic Acid	L-Arginine	2.03
172	Crocin III	Dimethyl fumarate	Orientin	1-Palmitoyl-sn-glycero-3-phosphocholine	2.04
173	3,4-Dimethoxyphenethylamine	Vanillyl Alcohol	Apigenin-7-glucuronide	Uridine 5'-monophosphate disodium salt	2.14
174	Cryptochlorogenic acid	Oleanolic Acid	Perillartine	8-Aminooctanoic acid	2.00
175	NADP disodium salt	Cholesteryl Acetate	Cynarin	D-Serine	2.05
176	D(-)-2-Aminobutyric acid	Lornoxicam	Alnustone	ATP disodium salt	2.15
177	Oxytetracycline Hydrochloride	Enrofloxacin	2-Pentylfuran	H-HoArg-OH	2.12

178	Arnidiol	Tripterin	Nootkatone	DL-Glutamine	0.26
179	methyl 2-hydroxy-4-methylvalerate	Ginsenoside Rc	alpha-Cyperone	Nepsilon-Acetyl-L-lysine	2.11
180	Cholesteryl oleate	3-(4-Hydroxyphenyl)-1-propanol	Casticin	Glycyl-L-valine	1.88
181	Pyridoxylamine	Rhapontigenin	Bergaptol	H-Tyr(3-I)-OH	2.13
182	Ergostenol	Ursolic acid acetate	2,5-Dihydroxyacetophenone	L-Alanyl-L-glutamine	1.95
183	Ethanolamine hydrochloride	Nomilin	Carvacrol	Aminomalonic acid	2.17
184	D-Leucine	Monotropein	Soyasaponin Bb	L-Leucyl-L-alanine	2.04
185	1,3-Dithiane	Linalool	Ginsenoside Rf	L-Leucine	2.06
186	1-Hydroxypyrene	1-Naphthaleneacetic acid	Sequoyitol	Glycyl-L-leucine	1.97
187	3,4-Dimethylbenzoic acid	Notoginsenoside Fe	dencichine	DMSO	2.08
188	2,4-Dihydroxybenzaldehyde	Dihydrokavain	Protodioscin	DMSO	2.13
189	2,5-Dihydroxybenzaldehyde	Ethyl maltol	3-Butylidenephthalide	DMSO	2.08
190	Allocholic acid	3-O-Methylgalangin	Eriodictyol	DMSO	2.04
191	1-Palmitoyl-2-oleoyl-sn-glycero-3-PC	Spermidine trihydrochloride	Nepitrin	DMSO	2.05
192	5-Methoxytryptophol	4-Ethyloctanoic acid	4,5-Dicaffeoylquinic acid	DMSO	2.05
193	11-Methoxyyangonin	trans-trans-Muconic acid	Ethyl palmitate	DMSO	2.13
194	5-Methoxyindole-3-acetic acid	Myosmine	N-trans-Feruloyltyramine	DMSO	2.07
195	Carveol	3b-Hydroxy-5-cholenoic acid	AKBA	DMSO	2.07
196	Methyl oleanonate	Bisphenol A	Caftaric acid	DMSO	2.20
197	Alashinol G	Methyl p-tert-butylphenylacetate	nerolidol	DMSO	2.20
198	Ganoderic acid B	Alloxan Monohydrate	N-Benzylpalmitamide	DMSO	2.20

199	L-Azetidine-2-carboxylic acid	Lindleyin	Araloside A	DMSO	1.98
200	Lignoceric Acid	NNK	kuwanon G	DMSO	2.15
201	4-Hydroxybenzylamine	DAPI Dihydrochloride	Ganoderic acid A	DMSO	2.01
202	Sulcatone	Sulforhodamine 101	Pyrogallol	DMSO	1.99
203	3-(3,4,5-Trimethoxyphenyl)propanoic acid	Fluorofenidone	Pulegone	DMSO	2.05
204	Piperonylic acid	10-Undecen-1-ol	Cryptochlorogenic acid	DMSO	2.02
205	D-(+)-Malic acid	Guanidinoethyl sulfonate	Methyl eugenol	DMSO	2.14
206	2-(Methylamino)-1H-purin-6(7H)-one	DL-Norvaline	Isoeugenol acetate	DMSO	2.07
207	8-Hydroxy-2'-deoxyguanosine	Fexofenadine	Ganoderic acid G	DMSO	2.01
208	Palmitelaidic Acid	Acetoxyvalerensre	Valepotriate	DMSO	1.40
209	α-Humulene	p-Cresyl sulfate	Pogostone	DMSO	1.94
210	1-Methylguanosine	N-Glycolylneuraminic acid	Isocurcumenol	DMSO	2.06
211	3-Methyluridine	2-Acetonaphthone	Furanodienone	DMSO	1.99
212	Se-Methylselenocysteine	Biotin sulfone	Avicularin	DMSO	1.91
213	Spinacine	Phenylalanylalanine	Notoginsenoside R2	DMSO	2.03
214	2-Hydroxy-4-methylbenzaldehyde	Lucideric acid A	Salvigenin	DMSO	1.98
215	Dydrogesterone	Beta-Tocopherol	Silydianin	DMSO	2.02
216	DL-Tryptophan	Biocytin	Ginsenoside F3	DMSO	2.02
217	δ-Tocotrienol	Hentriacontane	Vicenin 2	DMSO	1.92
218	N-Oleoyl glycine	Allyl Methyl Sulfide	Calenduloside E	DMSO	2.08
219	2,3-Butanediol	2-Phenylglycine	Hederasaponin B	DMSO	2.05
220	Questiomycin A	Nitrosoglutathione	Bilobetin	DMSO	1.86
221	Hydroxyphenyllactic acid	3-METHOXY-DL-TYROSINE	Notoginsenoside Fa	DMSO	2.12

222	1-Methylinosine	2-hydroxymethyl benzoic acid	Notoginsenoside Fc	DMSO	2.12
223	12-Ketodeoxycholic acid	Glutamyl-glutamic acid	Piperlongumine	DMSO	2.05
224	(Ethoxymethyl)benzene	4-Nitroquinoline 1-oxide	Agnuside	DMSO	2.03
225	Meconin	3-(IMIDAZOL-4-YL)PROPIONIC ACID	Artemitin	DMSO	1.93
226	2-Methylcyclohexanone	DL-2-AMINOOCTANOIC ACID	Thymoquinone	DMSO	1.98
227	2,2,2-Trichloroethanol	Methyl Undecenate	Deltonin	DMSO	1.74
228	cis-9,10-Epoxystearic acid	Jalapinolic acid	Esculentoside H	DMSO	2.10
229	Lathosterol	Ethyl phenylacetate	Auraptene	DMSO	2.05
230	1-Tetradecanol	Dimethyl succinate	Goserelin acetate	DMSO	1.94
231	Ethoxyacetic acid	Succinic anhydride	Anacardic Acid	DMSO	2.00
232	α-Tocotrienol	Sucrose octaacetate	D-ERYTHRO-SPHINGOSINE	DMSO	2.02
233	Stigmastanol	(+)-Bornyl Acetate	Fucosterol	DMSO	2.03
234	2,4-Di-tert-butylphenol	N-Valyltryptophan	Oxfendazole	DMSO	2.02
235	Thioacetamide	6-Phosphogluconic acid	Tributyrin	DMSO	2.07
236	Soyasaponin Ab	2-Methoxyestrone	Taurochenodeoxycholic Acid	DMSO	1.99
237	Neopterin	Omeprazole metabolite Omeprazole sulfone	D-(+)-Trehalose	DMSO	2.17
238	Ganoderic acid F	Poloxamer 407	Imidazole	DMSO	2.13
239	1-Naphthol	Methyl cellulose	DMSO	DMSO	2.05
240	Kuwanon H	2-ATHBI	DMSO	DMSO	2.10
241	DMSO	DMSO	DMSO	DMSO	2.00

Table S2 Effects of 48 compounds on SeV-induced ISRE activity

Compound	Rel. activity of ISRE (log_{10} %)
Amcinonide	1.87
Cyclamic acid	1.89
Cucurbitacin B	0.09
Gonadorelin Acetate (33515-09-2 free base)	1.91
Tetracycline	1.91
Arctigenin	1.77
Palmitoylethanolamide	1.87
cis,cis-Muconic acid	1.06
Docetaxel	1.65
Costunolide	1.65
N6-methyladenosine	1.99
Maltol	1.90
Geniposidic Acid	1.82
Sanguinarine	0.04
Didymin	1.95
Tiglic acid	2.03
Ascorbyl palmitate	1.88
Harmine	1.32
Isomangiferin	1.91
2-Hydroxy-2-methylbutanoic acid	1.89
Methyldopa	1.93
Rapamycin	1.66
Vitamin D3	1.84
TBHQ	1.80
Dinitolmide	1.89
Levobupivacaine	1.90
Acevaltrate	0.59
Fenchyl Alcohol	1.96
Fenbendazole	1.68
Ginsenoside Re	2.11
2,6-Dimethoxybenzoic acid	2.04
7,4'-Di-O-methylapigenin	2.10
Delta-Tocopherol	1.98
medicagenic acid	1.89
physalin F	0.04
1-Eicosanol	1.90
Terpinen-4-ol	1.98
Diallyl Trisulfide	2.10
11-Keto-beta-boswellic acid	2.04
4-Hydroxynonenal	0.62
Arnidiol	1.92

Tripterin	-0.11
Nootkatone	1.97
DL-Glutamine	1.88
Palmitelaidic Acid	1.97
Acetoxyvalerensre	1.88
Valepotriate	0.29
N-Oleoyl glycine	1.93
DMSO	2.00

Table S3 A list of candidate proteins targeted by physalin F from LiP-MS

Protein ID	No. Diff. Peptides	Abs. Log2 FC	No. Anno. Terms	PPI Degree	No. Function Types	Docking Affinity	Score
P10809	4	1.9	64	82	1	-8.537	10016.1
Q02880	11	1.6	18	26	1	-10.56	9302.5
P68104	3	2.2	31	72	0	-10.335	6884.4
P68032	2	6.8	48	8	0	-8.573	6514.6
P04179	1	4.5	91	50	1	-9.009	5873.3
P00390	4	1.3	60	38	1	-9.596	5169.6
Q9Y4W6	3	2.4	35	46	0	-8.849	5087.4
P11388	5	1.0	25	54	1	-10.056	4265.8
P12956	1	6.0	25	42	0	-10.457	4210.4
P04844	5	1.5	11	28	1	-9.36	3146.6
P06576	2	1.0	50	80	0	-9.358	2472.7
Q9HCU5	2	3.4	15	18	0	-10.405	2311.4
Q00610	2	1.4	26	48	0	-10.442	2228.2
P39656	4	1.1	20	34	1	-8.582	2157.3
Q9Y617	1	7.4	18	14	0	-8.815	2090.6
P07437	2	1.4	35	48	0	-8.593	2033.3
P63261	2	1.6	43	36	0	-7.964	2028.1
P05141	2	1.3	47	24	1	-10.028	1990.1
Q00059	2	1.4	32	44	1	-8.276	1925.0
P07900	1	1.1	55	104	1	-9.162	1666.8

Score= -["No. Diff. Peptides" * "Abs. Log2 FC" * ("No. Anno. Terms" + "PPI Degree" + 5 * "No. Function Types") * "docking_affinity"]

Figure S1

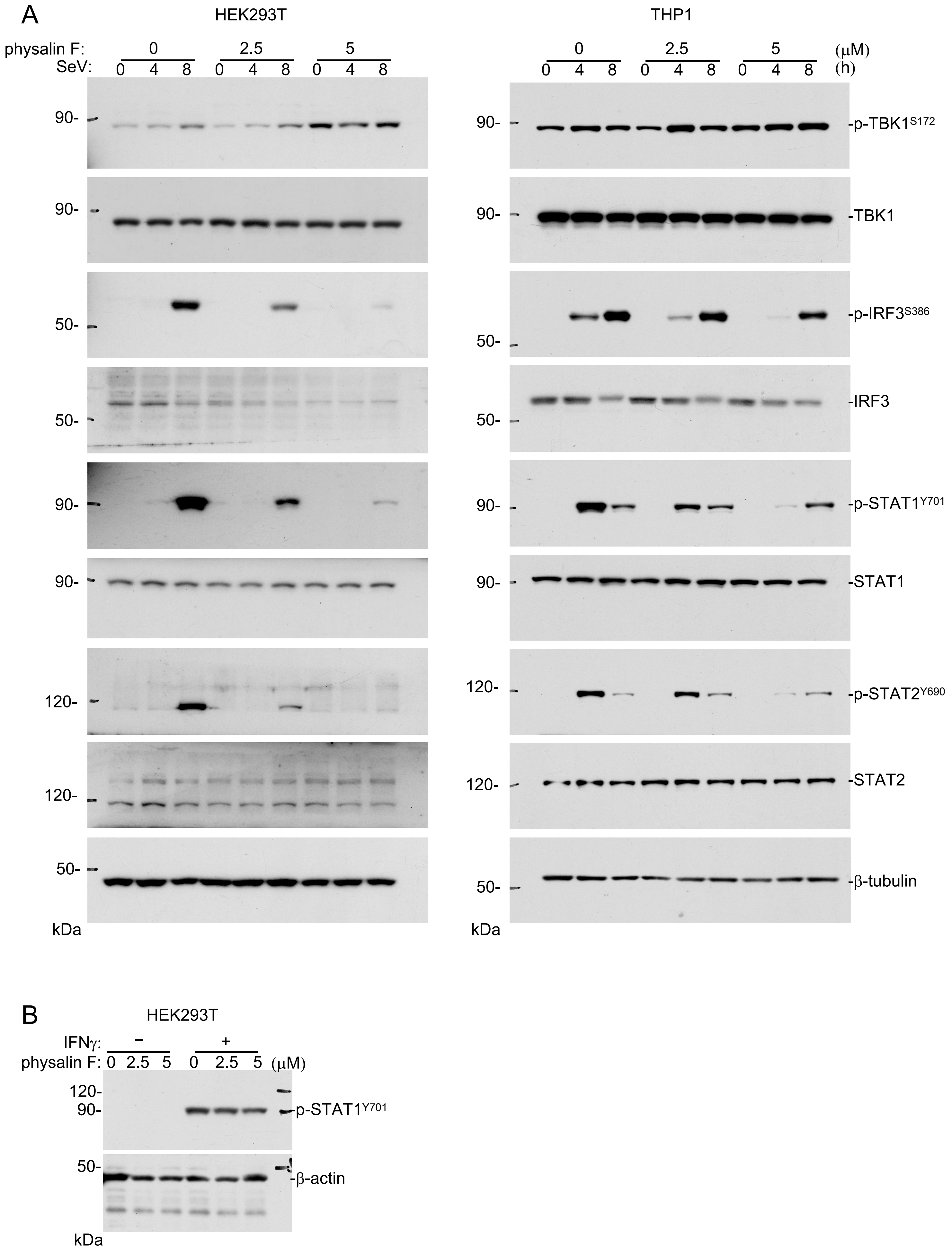
A
HEK293T
THP1
physalin F: 0 2.5 5 (μM)
SeV: 0 4 8 0 4 8 0 4 8 (h)
90-
50-
120-
kDa
-p-TBK1S172
-TBK1
-p-IRF3S386
-IRF3
-p-STAT1Y701
-STAT1
-p-STAT2Y690
-STAT2
-β-tubulin
B
HEK293T
IFNγ: – +
physalin F: 0 2.5 5 0 2.5 5 (μM)
120-
90-
50-
-p-STAT1Y701
-β-actin
kDa

Figure S2

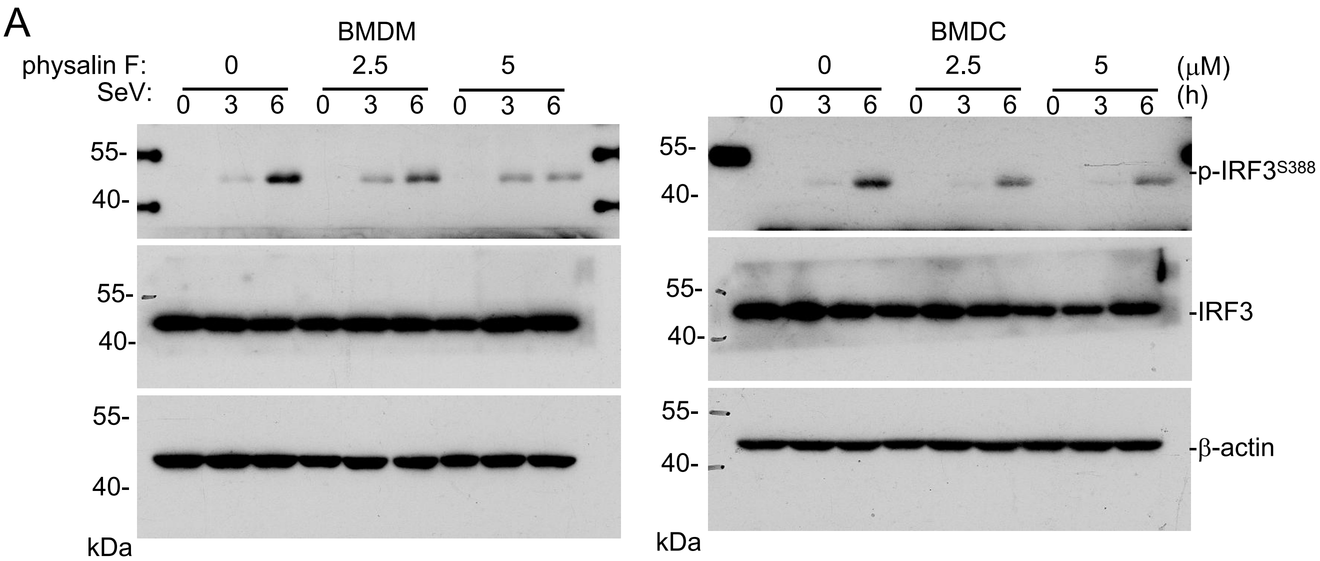
A
BMDM
BMDC
physalin F:
SeV:
0 2.5 5
0 3 6 0 3 6 0 3 6
(μM)
(h)
55-
40-
kDa
-p-IRF3S388
-IRF3
-β-actin

Figure S3

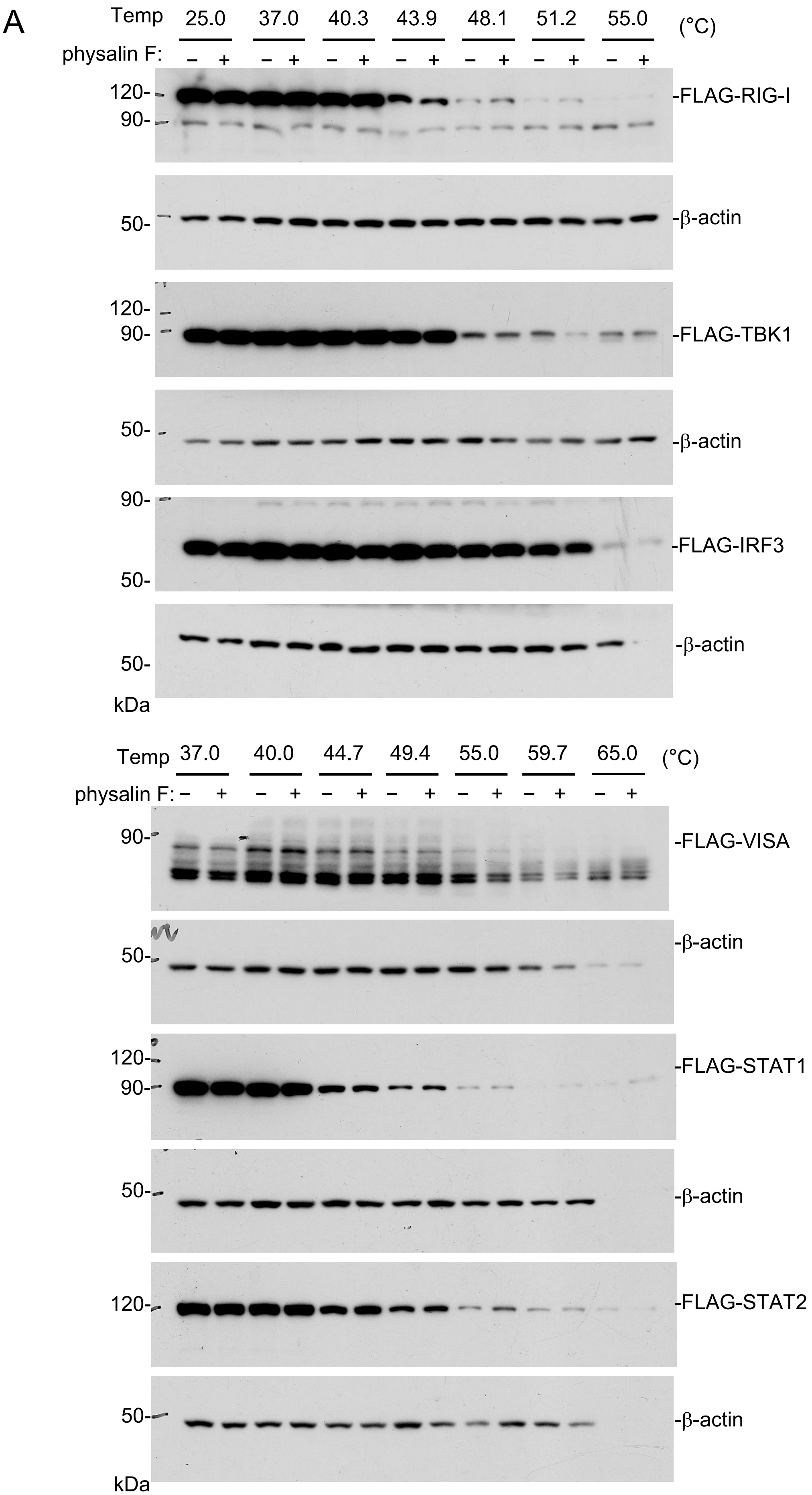
A
Temp 25.0 37.0 40.3 43.9 48.1 51.2 55.0 (°C)
physalin F: − + − + − + − + − + − + − +
120-
90-
-FLAG-RIG-I
50-
-β-actin
120-
90-
-FLAG-TBK1
50-
-β-actin
90-
-FLAG-IRF3
50-
-β-actin
50-
kDa
Temp 37.0 40.0 44.7 49.4 55.0 59.7 65.0 (°C)
physalin F: − + − + − + − + − + − + − +
90-
-FLAG-VISA
50-
-β-actin
120-
90-
-FLAG-STAT1
50-
-β-actin
120-
-FLAG-STAT2
50-
-β-actin
kDa

Figure S4

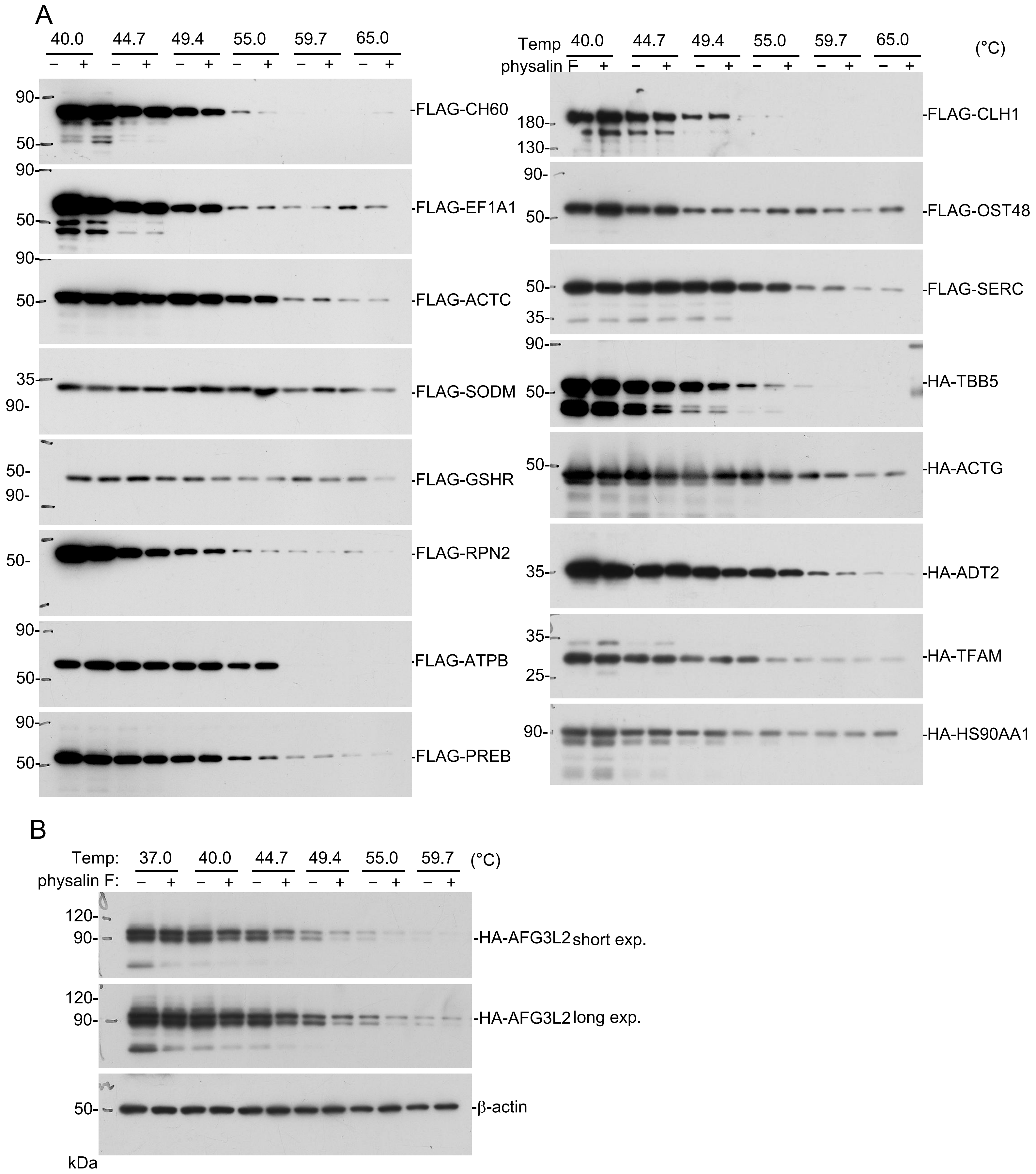
A
Temp 40.0 44.7 49.4 55.0 59.7 65.0 (°C)
physalin F – + – + – + – + – + – +
90- 50- -FLAG-CH60
90- 50- -FLAG-EF1A1
90- 50- -FLAG-ACTC
35- -FLAG-SODM
90- 50- -FLAG-GSHR
90- 50- -FLAG-RPN2
90- 50- -FLAG-ATPB
90- 50- -FLAG-PREB
180- 130- -FLAG-CLH1
90- 50- -FLAG-OST48
50- 35- -FLAG-SERC
90- 50- -HA-TBB5
50- -HA-ACTG
35- -HA-ADT2
35- 25- -HA-TFAM
90- -HA-HS90AA1
B
Temp: 37.0 40.0 44.7 49.4 55.0 59.7 (°C)
physalin F: – + – + – + – + – + – +
120- 90- -HA-AFG3L2 short exp.
120- 90- -HA-AFG3L2 long exp.
50- -β-actin
kDa

Figure S5

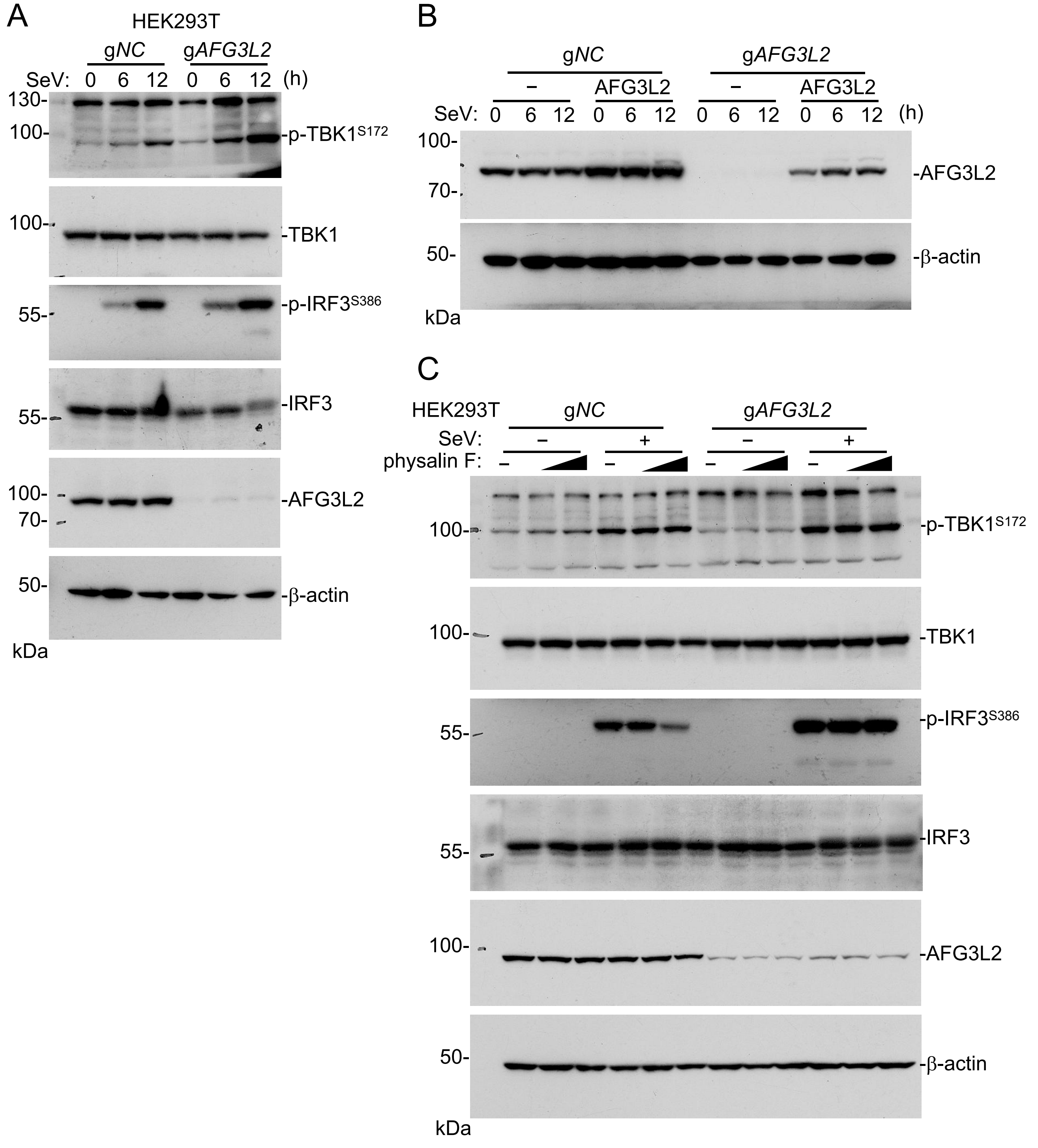

A
HEK293T
gNC
gAFG3L2
SeV: 0 6 12 0 6 12 (h)
130-
100-
-p-TBK1S172
100-
-TBK1
55-
-p-IRF3S386
55-
-IRF3
100-
70-
-AFG3L2
50-
-β-actin
kDa
B
gNC
gAFG3L2
–
AFG3L2
–
AFG3L2
SeV: 0 6 12 0 6 12 0 6 12 0 6 12 (h)
100-
70-
-AFG3L2
50-
-β-actin
kDa
C
HEK293T
gNC
gAFG3L2
SeV:
–
+
–
+
physalin F:
–
–
–
–
100-
-p-TBK1S172
100-
-TBK1
55-
-p-IRF3S386
55-
-IRF3
100-
-AFG3L2
50-
-β-actin
kDa

Figure S6

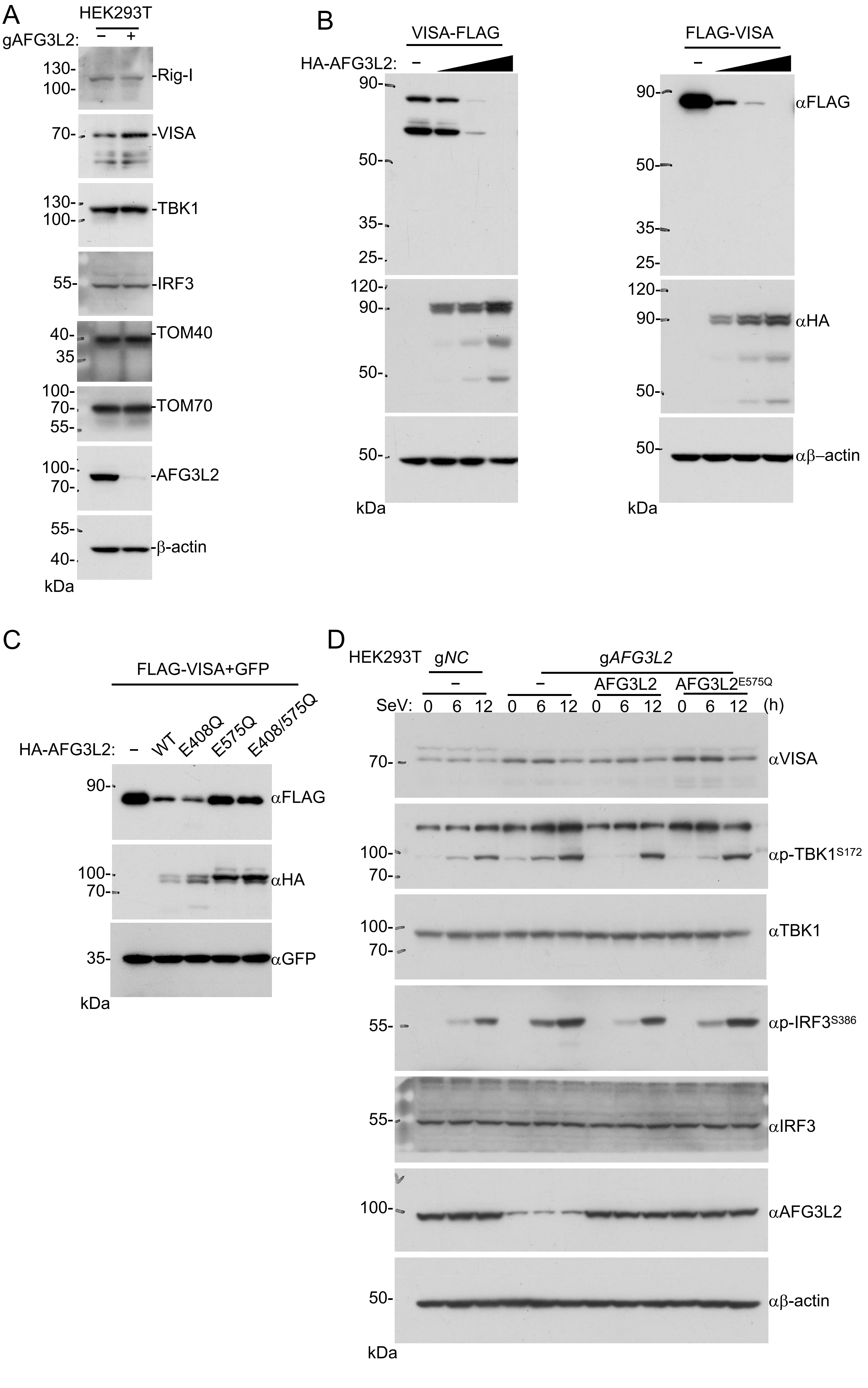
A
HEK293T
gAFG3L2:
−
+
130-
100-
-Rig-I
70-
-VISA
130-
100-
-TBK1
55-
-IRF3
40-
35
-TOM40
100-
70-
55-
-TOM70
100-
70-
-AFG3L2
55-
40-
-β-actin
kDa
B
VISA-FLAG
HA-AFG3L2:
−
90-
50-
35-
25-
120-
90-
50-
50-
kDa
FLAG-VISA
−
90-
50-
35-
25-
αFLAG
120-
90-
50-
αHA
50-
αβ–actin
kDa
C
FLAG-VISA+GFP
HA-AFG3L2:
−
WT
E408Q
E575Q
E408/575Q
90-
αFLAG
100-
70-
αHA
35-
αGFP
kDa
D
HEK293T
gNC
gAFG3L2
−
−
AFG3L2
AFG3L2E575Q
SeV:
0
6
12
0
6
12
0
6
12
0
6
12
(h)
70-
αVISA
100-
70-
αp-TBK1S172
100-
70-
αTBK1
55-
αp-IRF3S386
55-
αIRF3
100-
αAFG3L2
50-
αβ-actin
kDa

Figure S7

A

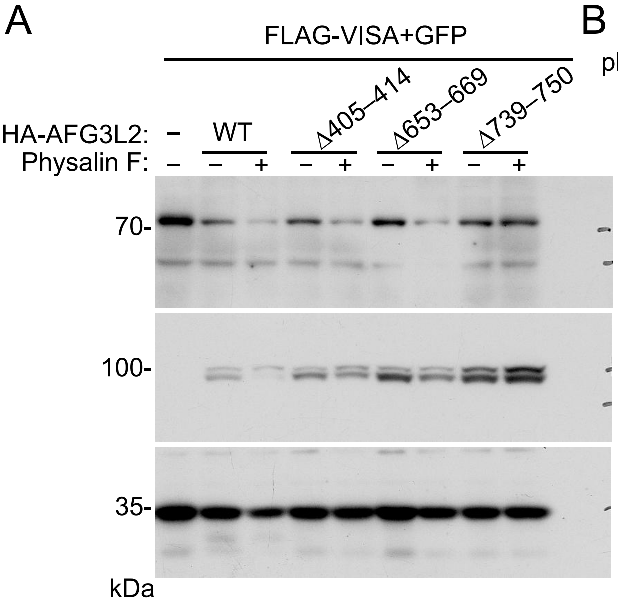
FLAG-VISA+GFP
HA-AFG3L2: – WT Δ405–414 Δ653–669 Δ739–750
Physalin F: – – + – + – + – +
70-
100-
35-
kDa

B

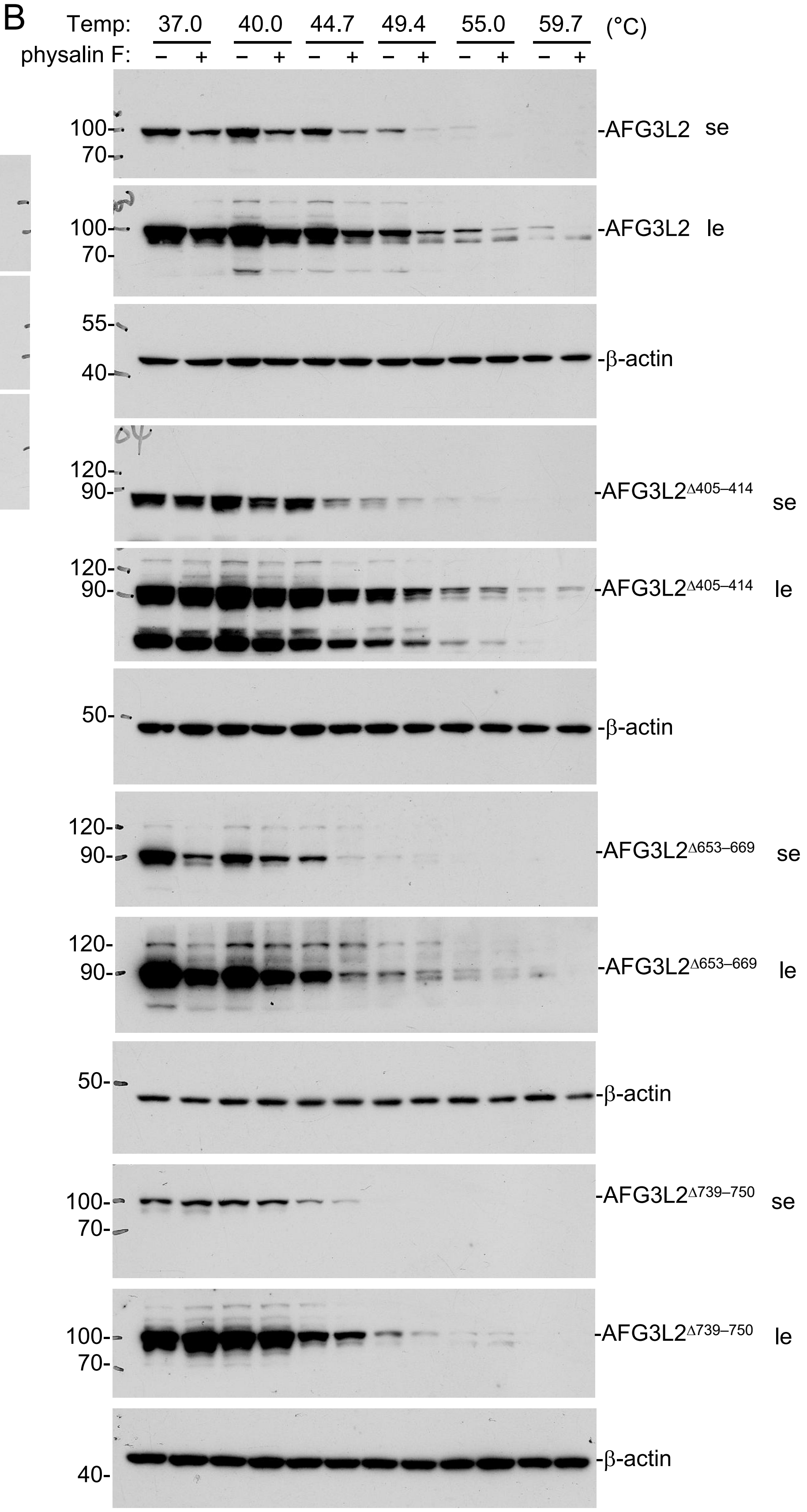
Temp: 37.0 40.0 44.7 49.4 55.0 59.7 (°C)
physalin F: – + – + – + – + – + – +
100-
70-
-AFG3L2 se
-AFG3L2 le
55-
40-
-β-actin
120-
90-
-AFG3L2Δ405–414 se
-AFG3L2Δ405–414 le
50-
-β-actin
-AFG3L2Δ653–669 se
-AFG3L2Δ653–669 le
-β-actin
-AFG3L2Δ739–750 se
-AFG3L2Δ739–750 le
-β-actin
